# Sex-Hormone-Binding Globulin Gene Polymorphisms and Breast Cancer Risk in Caucasian Women of Russia

**DOI:** 10.3390/ijms25042182

**Published:** 2024-02-11

**Authors:** Irina Ponomarenko, Konstantin Pasenov, Maria Churnosova, Inna Sorokina, Inna Aristova, Vladimir Churnosov, Marina Ponomarenko, Evgeny Reshetnikov, Mikhail Churnosov

**Affiliations:** Department of Medical Biological Disciplines, Belgorod State National Research University, 308015 Belgorod, Russia; ponomarenko_i@bsu.edu.ru (I.P.); 944472@bsu.edu.ru (K.P.); churnosovamary@gmail.com (M.C.); sorokina@bsu.edu.ru (I.S.); aristova@bsu.edu.ru (I.A.); 958561@bsu.edu.ru (V.C.); 1256888@bsu.edu.ru (M.P.); reshetnikov@bsu.edu.ru (E.R.)

**Keywords:** SHBG, breast cancer, SNP, association

## Abstract

In our work, the associations of GWAS (genome-wide associative studies) impact for sex-hormone-binding globulin (SHBG)-level SNPs with the risk of breast cancer (BC) in the cohort of Caucasian women of Russia were assessed. The work was performed on a sample of 1498 women (358 BC patients and 1140 control (non BC) subjects). SHBG correlated in previously GWAS nine polymorphisms such as rs780093 *GCKR*, rs17496332 *PRMT6*, rs3779195 *BAIAP2L1*, rs10454142 *PPP1R21*, rs7910927 *JMJD1C*, rs4149056 *SLCO1B1*, rs440837 *ZBTB10*, rs12150660 *SHBG*, and rs8023580 *NR2F2* have been genotyped. BC risk effects of allelic and non-allelic SHBG-linked gene SNPs interactions were detected by regression analysis. The risk genetic factor for BC developing is an SHBG-lowering allele variant C rs10454142 *PPP1R21* ([additive genetic model] OR = 1.31; 95%CI = 1.08–1.65; p_perm_ = 0.024; power = 85.26%), which determines 0.32% of the cancer variance. Eight of the nine studied SHBG-related SNPs have been involved in cancer susceptibility as part of nine different non-allelic gene interaction models, the greatest contribution to which is made by rs10454142 *PPP1R21* (included in all nine models, 100%) and four more SNPs—rs7910927 *JMJD1C* (five models, 55.56%), rs17496332 *PRMT6* (four models, 44.44%), rs780093 *GCKR* (four models, 44.44%), and rs440837 *ZBTB10* (four models, 44.44%). For SHBG-related loci, pronounced functionality in the organism (including breast, liver, fibroblasts, etc.) was predicted in silico, having a direct relationship through many pathways with cancer pathophysiology. In conclusion, our results demonstrated the involvement of SHBG-correlated genes polymorphisms in BC risk in Caucasian women in Russia.

## 1. Introduction

Epidemiological data obtained by the International Agency for Research on Cancer based on a study of 36 different tumors in 185 countries of the world show that in 2020, more than 2.261 million new cases of BC (11.7% of all cancer cases) and almost 685 thousand deaths from this disease (6.9% of all cases) were registered worldwide [1,2]. According to the Global Cancer Observatory, in 2020, the incidence of BC in the world was 47.8 per 100 thousand population, and mortality from this disease was 13.6 per 100 thousand population [3]. BC is the most common cancer (24.5%) and the most common cause of cancer death (15.5%) in women [1,2]. Over the next 20 years (from 2020 to 2040), the WHO (Global Cancer Observatory data) predicts a significant increase in the number of women with BC (by 39%, from 2.3 million to 3.2 million) and deaths from BC (by 47%, from 0.68 million to 1.00 million) [3].

BC from genetic positions (twin, family, associative, and GWAS studies) has been actively studied in recent decades [4,5,6,7,8,9,10,11]. Considerable factual material has been accumulated on this theme, convincingly showing the strong contribution of hereditary factors to BC susceptibility [4,8]. Firstly, according to large-scale twin studies performed in European populations, and including materials on several tens [5] and hundreds [6] of thousands of twin pairs, the contribution of “genetics” to BC development is 31%. Secondly, up to 25% of hereditary cases of the disease are caused by mutations in highly penetrant (*BRCA1*, *BRCA2*, *PTEN*, *TP53*, *CDH1*, *STK11*) (increase the risk of developing BC by up to 80%) and 2–3% in moderately penetrant (*CHEK2*, *BRIP1*, *ATM*, *PALB2*) (cause a 2-fold increased BC risk) genes [4]. Thirdly, the results of large-scale GWAS showed associations with the disease of over 220 polymorphic loci of numerous candidate genes [7,8,9,10], and these GWAS SNPs “explain” 18% of the heritability of BC [7]. However, at the same time, only 30–40% patients with BC have a burdened family history, and only 5% cases of the disease are associated with mutations in highly and moderately penetrant genes [8]. Furthermore, GWAS loci “describe” only about 44% of the putative genetic determinants of BC (18% of 41%) [7], which indicates the presence of a problem of “hidden” heredity in BC, and determines the relevance of further genetic studies of the disease.

BC is a hormone-dependent disease, in the pathophysiology of which sex hormones (estrogens, testosterone, progesterone, etc.) are widely “involved” [12,13,14,15]. It is believed that a higher level of sex hormones increases the risk of BC developing; however, these relationships (presence/orientation) depend on the pre/postmenopausal status, a woman’s body mass index (BMI), the receptor status of the tumor, etc. [13,14,16]. The pathophysiological links between steroids and BC depend on SHBG, as this protein is involved in regulating the concentration of bioavailable testosterone and estradiol [17,18,19,20].

SHBG (glycoprotein has a mass of 90–100 kDa) is synthesized mainly in the liver, consists of two identical peptide chains, and contains “specific” sex hormone binding sites [18]. Due to the presence of these sites, SHBG “binds” steroid hormones (testosterone, estrogens) and, as a result, transports them. At the same time, steroids associated with SHBG do not show their biological activity, and only “free” (not related to SHBG) sex hormones are active and realize their biological effects in the organism. It is believed that a significant part of testosterone (65%) and estrogens (estradiol) (38%) in the organism is in a SHBG-related state, and only small amounts of them (about 1–2% of testosterone and 2% of estradiol) are biologically active (not related to SHBG) [19]. Thus, SHBG is a very important “regulator” of the level and, accordingly, the activity of sex hormones (testosterone, estrogens) in the body (a high level of SHBG leads to a low content of free steroids, and, accordingly, a low level of SHBG causes an increase in the concentration of bioavailable sex hormones), which may be of paramount importance for the pathophysiology of BC [13,14,16,21].

Previously performed genetic studies have established a significant contribution of hereditary factors to the determination of circulating SHBG levels in women (56–58%) [17,22]. Using the Mendelian randomization (MR) method, it has been shown that, in the genetic correlation of SNPs and GWAS associated with the level of SHBG (determining ~8.4% of the genetic variability of the SHBG concentration in women) [23] with the risk of BC, the orientation of these associations (risk/protective) directly depended on the receptor status of the tumor [24]. At the same time, it should be noted that the results of numerous previous studies devoted to the study of the role of individual genetic determinants of SHBG in BC formation are ambiguous [25,26,27,28,29,30,31,32,33], and the contribution of individual GWAS significant for SHBG loci to BC susceptibility has not been studied; this determines the relevance of this work. So then, our work assessed the associations of GWAS impact for SHBG-level loci with BC risk in the cohort of Caucasian women in Russia.

## 2. Results

In BC and non-BC subjects, the registered genotypes’ distribution entirely matched with the anticipated distribution, according the parameters of the Hardy–Weinberg rule (p_bonf_ > 0.006 [0.05/9]. When evaluating the data, the Bonferroni amendment was implemented for the number of loci studied [n = 9]) (Supplementary Table S1).

Among the nine SHBG-impacted loci considered, one SNP—rs10454142 *PPP1R21*—showed a correlation with BC (Table 1). Minor allele C rs10454142 *PPP1R21*, being in the woman genotype, raised the risk of BC by 15–16% for each allele (CC vs. TC vs. TT [additive model]; OR = 1.31; 95%CI = 1.08–1.65; *p* = 0.022; p_perm_ = 0.024; power = 85.26%).

As a result of the evaluation of the multi-locus BC risk effects of the nine studied SNPs, the nine most significant models of SHBG-related gene interlocus interactions were obtained (Table 2). Importantly, the levels of statistical significance actually used by us for the selection of different levels models were higher than the values that we set as “threshold” at the beginning of this study, and in particular, for the selection of two SNP models, we actually used a level equal to *p* < 1.23 × 10^−4^ (an order of magnitude higher than the one established at the beginning, the “threshold” level is equal to *p* = 1.39 × 10^−3^), for three SNP models—*p* < 1.64 × 10^−6^ (more than two orders of magnitude higher than the originally set “threshold” level—*p* = 5.95 × 10^−4^), for four SNP models—*p* < 4.65 × 10^−9^ (five orders of magnitude higher than the initially accepted “threshold” value—*p* = 3.97 × 10^−4^), for five SNP models—*p* < 4.79 × 10^−12^ (almost eight orders of magnitude higher than the indicator originally set—*p* = 3.97 × 10^−4^) (Table 2). This indicates extremely minimal risks of obtaining false-positive results, and allows us to speak about the reliability and high statistical significance of the results of interlocus modeling obtained by us associated with the risk of BC. It is also important to note that the simulation results were validated by us using permutation testing at the level of permutation ≤ 0.001, and at that all nine models corresponded to this threshold level.

Of the nine studied loci, eight polymorphisms of SHBG-related genes were part of the BC-risk interlocus models, such as rs780093 *GCKR*, rs17496332 *PRMT6*, rs3779195 *BAIAP2L1*, rs10454142 *PPP1R21*, rs7910927 *JMJD1C*, rs4149056 *SLCO1B1*, rs440837 *ZBTB10*, and rs8023580 *NR2F2* (Table 2). One locus, rs12150660 *SHBG*, was not involved in disease susceptibility either independently or as part of SNP interaction models. All nine significant models include rs10454142 *PPP1R21*, which previously showed independent strong associations with BC. Herewith, four more loci were part of more than 40% of all significant models: rs7910927 *JMJD1C* (five models), rs17496332 *PRMT6* (four models), rs780093 *GCKR* (four models), and rs440837 *ZBTB10* (four models), which indicates their essential role in the BC formation (Table 2). The two-locus combination rs440837 *ZBTB10*-rs10454142 *PPP1R21* was the basis for four BC-impacted models (44.44%), and the other two-locus combination, rs4149056 *SLCO1B1*-rs10454142 *PPP1R21*, was part of three BC-associated models (33.33%).

We have obtained two five-locus models of SNP interactions of SHBG-related genes showing the most pronounced effect in relation to BC susceptibility (Table 2): rs7910927 *JMJD1C*-rs3779195 *BAIAP2L1*-rs10454142 *PPP1R21*-rs780093 *GCKR*-rs17496332 *PRMT6* (Wald index for risky combinations of this model was the maximum and equals 51.58) and rs8023580 *NR2F2*-rs7910927 *JMJD1C*-rs10454142 *PPP1R21*-rs780093 *GCKR*-rs17496332 *PRMT6* (the Wald index for risky combinations of this model is also the highest and equals 47.77). Interestingly, the indicators of statistical significance of these two models (*p* = 6.88 × 10^−13^ and *p* = 4.78 × 10^−12^, respectively, with p_perm_ < 0.001 for both models) significantly exceed the threshold values used in GWAS (*p* ≤ 5 × 10^−8^).

The calculations revealed 44 different combinations of genotypes within the framework of 9 interaction models that were highly relevant to the BC risk (Supplementary Table S2), among which more than 84% had a risk orientation (34/44; 84.09%) and only about 16% had a BC-protective value (7/44; 15.91%). The most expressed BC-risk phenotypic effects (they are distinguished by highest values of the regression coefficients (*beta*)) had such genotypes combinations as rs7910927-GT *JMJD1C*-rs3779195-TA *BAIAP2L1*-rs10454142-CC *PPP1R21*-rs780093-CT *GCKR*-rs17496332-AA *PRMT6* (*beta* = 3.926/*p* = 0.0003), rs7910927-GT *JMJD1C*-rs10454142-CC *PPP1R21*-rs780093-CC *GCKR*-rs17496332-AA *PRMT6* (*beta* = 3.926/*p* = 0.0003), rs4149056-CC *SLCO1B1*-rs440837-AA *ZBTB10*-rs10454142-CC *PPP1R21*-rs780093-CT *GCKR* (*beta* = 3.756/*p* = 0.016), rs4149056-CC *SLCO1B1*-rs440837-AA *ZBTB10*-rs10454142-CC *PPP1R21* (*beta* = 3.756/*p* = 0.016) (combinations of “risky” orientation); rs8023580-TC *NR2F2*-rs7910927-GG *JMJD1C*-rs10454142-TC *PPP1R21*-rs780093-CT *GCKR*-rs17496332-AG *PRMT6* (*beta* = −2.202/*p* = 0.048), rs7910927-GT *JMJD1C*-rs3779195-TT *BAIAP2L1*-rs10454142-TC *PPP1R21*-rs780093-CC *GCKR*-rs17496332-AA *PRMT6* (*beta* = −1.827/*p* = 0.007), and rs7910927-GT *JMJD1C*-rs10454142-TC *PPP1R21*-rs780093-CC *GCKR*-rs17496332-AA *PRMT6* (*beta* = −1.827/*p* = 0.007) (combinations of “protective” orientation) (Supplementary Table S2). The three-locus “risky” combination rs7910927-GT *JMJD1C*-rs440837-AA *ZBTB10*-rs10454142-CC *PPP1R21* (*beta* = 1.586) is associated with BC with the greatest statistical significance (*p* = 0.00002).

**Figure 1:**
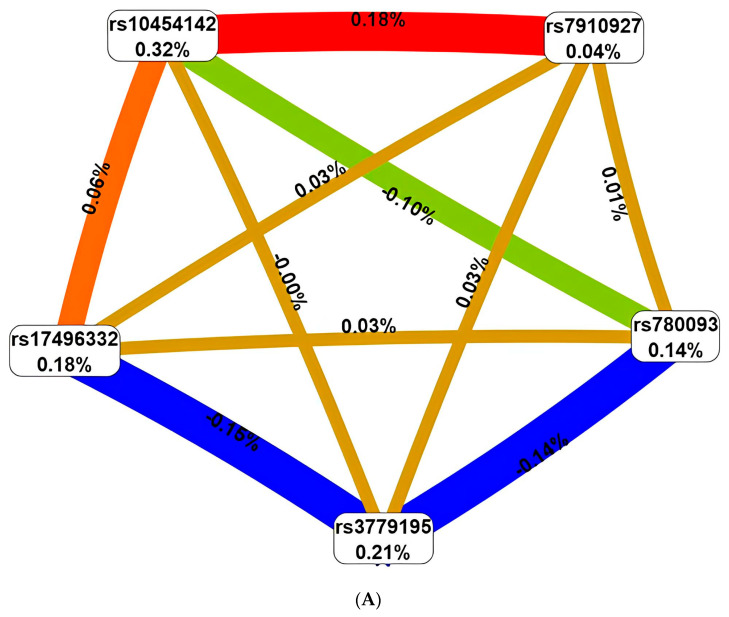

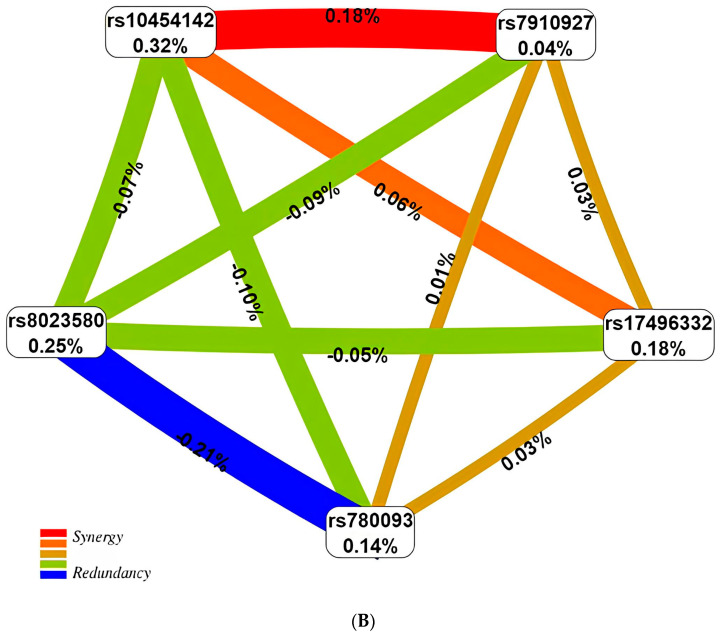
The entropy graph of the five loci models of SNP × SNP interactions associated with breast cancer based on the MDR analysis: Model 1 (**A**) (Wald st. = 51.58, *p* = 6.88 × 10^−13^, p_perm_ < 0.001), Model 2 (**B**) (Wald st. = 47.77, *p* = 4.78 × 10^−12^, p_perm_ < 0.001). The red and orange colors denote strong and moderate synergism, respectively, brown color denotes the independent effect, green and blue colors denote moderate and strong antagonism, respectively.

**Figure 2:**
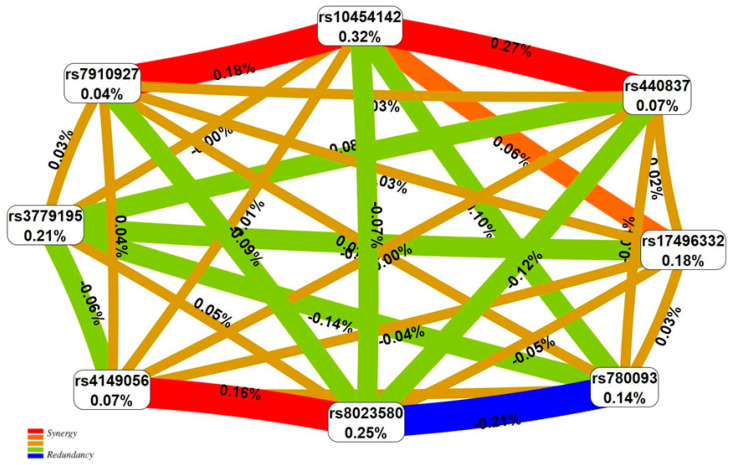
The entropy graph of the SNP × SNP interactions associated with breast cancer based on the MDR analysis. Positive values of entropy indicate synergistic interactions, while the negative values indicate redundancy. The red and orange colors denote strong and moderate synergism, respectively; brown color denotes the independent effect; green and blue colors denote moderate and strong antagonism.

We performed visualization in the form of SNP interaction graphs that determine the BC risk, both within the framework of the two very valuable for the disease of 5-locus models (Figure 1), and within all eight SNPs involved in BC susceptibility (Figure 2). For the 5-locus model rs7910927 *JMJD1C*-rs3779195 *BAIAP2L1*-rs10454142 *PPP1R21*-rs780093 *GCKR*-rs17496332 *PRMT6*, synergistic interactions with an entropy of 0.18% were found between rs7910927 *JMJD1C* and rs10454142 *PPP1R21*, and antagonistic interactions between rs3779195 *BAIAP2L1*, on the one hand, and loci rs780093 *GCKR* (entropy—−0.14%), rs17496332 *PRMT6* (entropy—−0.15%) on the other hand (Figure 1A). For another 5-locus model, rs8023580 *NR2F2*-rs7910927 *JMJD1C*-rs10454142 *PPP1R21*-rs780093 *GCKR*-rs17496332 *PRMT6*, the synergistic interaction of rs7910927 *JMJD1C*-rs10454142 *PPP1R21* (0.18%) was confirmed, and antagonistic interaction between rs8023580 *NR2F2* and rs780093 *GCKR* was found (entropy—−021%) (Figure 1B).

When considering the interactions of all eight SHBG-related loci correlated with BC risk, the following can be noted (Figure 2). Firstly, the contribution of rs10454142 *PPP1R21* to disease susceptibility (0.32%) is the highest among all other seven BC-significant loci (from 0.04% for rs7910927 *JMJD1C* to 0.25% for rs8023580 *NR2F2*) and their paired interactions (the highest estimates reach 0.27%). Secondly, rs10454142 *PPP1R21* synergistically interacts with two loci—rs440837 *ZBTB10* (0.27%) and rs10454142 *PPP1R21* (0.18%). Third, SNP rs8023580 *NR2F2* demonstrates antagonistic interactions with rs780093 *GCKR* (−0.21%) and synergistic interactions with rs4149056 *SLCO1B1* (0.16%).

Positive values of entropy indicate synergistic interactions, while the negative values indicate redundancy. The red and orange colors denote strong and moderate synergism, respectively; brown color denotes the independent effect; and green and blue colors denote moderate and strong antagonism.

### 2.1. Predicted Functionality of BC-Causal Loci

We have studied the supposed functional effects of BC-involved polymorphisms in 2 directions: (1) the functional significance of rs10454142 *PPP1R21* (and 10 SNPs strongly linked to it), which demonstrated the main risk effect for BC, was considered; (2) the functional potential of all 8 SHBG-related polymorphisms associated with BC risk was evaluated; (3) summary functionality of all 130 BC-correlated loci (8 BC-causal and 122 proxy SNPs) was examined.

The results of the first stage of the study devoted to an in-depth and detailed consideration of the functional effects of rs10454142 *PPP1R21* and 10 proxy polymorphisms are presented in Table 3 and Table 4, and Supplemental Table S3—Supplemental Table S6. It was found that, firstly, rs10454142 *PPP1R21* and two SNPs located next to it (rs201414717 and rs10454143), exhibit pronounced functional activity (they are localized in the area of enhancers/promoters, active enhancers/promoters) in the liver (the organ in which SHBG is mainly synthesized [18]) (Table 3).

Secondly, rs10454142 *PPP1R21* and five high-linked variants positioned in the area of three genes—*PPP1R21*, *FOXN2*, and *KLRAQ1*—are disposed in the province of enhancers/promoters in breast cell lines—Breast variant Human Mammary Epithelial Cells and Breast Myoepithelial Primary Cells (Table 3). So, the polymorphism rs10454142 PPP1R21, and the four SNPs in the linkage disequilibrium (LD) with it (rs78597273; rs11689645; rs201414717; rs10454143), have been located in the enhancer region in Breast variant Human Mammary Epithelial Cells (rs78597273 was additionally located in the enhancer region in Breast Myoepithelial Primary Cells), and another linked polymorphism, rs4638844, was placed in the active promoter region in Breast Myoepithelial Primary Cells.

Thirdly, rs10454142 *PPP1R21* and 8 SNPs in LD affect the interaction of regulatory DNA regions with 51 transcription factors (TFs) (AP-1, AP-2, AP-4, Bach1, Bach2, BAF155, Barx1, BATF, BCL, CIZ, CACD, CEBPD, ELF1, GR, FAC1, Fox, Foxa, Irf, Foxd3, Foxj2, Foxk1, Foxo, Foxp1, GATA, HDAC2, HMGN3, Hoxa3, Hoxa5, Hoxb6, KAP1, LBP-1, Maf, MIZF, Myc, NF-E2, NF-kappaB, p300, Sox, STAT, ZBRK1, Pax-4, PRDM1, Rad21, RREB-1, TAL1, TCF12, TCF4, WT1, YY1, ZNF219, Zfp105) (Table 3), distinguished by pronounced co-expression of several TFs such as HOXA3 and HOXA5 (co-expression score 0.756), HOXB6 and HOXA5 (co-expression score 0.489), EP300 and BPTF (co-expression score 0.423), RAD21 and YY1 (co-expression score 0.357), HOXB6 and HOXA3 (co-expression score 0.285), ZNF384 and FOXJ2 (co-expression score 0.226), and TRIM28 and SMARCC1 (co-expression score 0.201) (STRING data)). With the help of Gene Ontology Resource, about 300 different biological pathways have been identified in which the described above 51 TFs (Supplementary Table S3) are “involved”, among which the processes of gene transcription regulation have the greatest statistical significance (p(FRD) > 1 × 10^−15^), including due to cis-influences on the regulatory regions of DNA, modulation activity of RNA polymerase II, biosynthesis and metabolism of RNA, etc. Herewith, the following pathways have the maximum “overrepresentation” (Fold Enrichment (FE) index of more than 100): STAT3 nuclear events downstream of ALK signaling (R-HSA-9701898; p(FRD) = 6.40 × 10^−4^), positive regulation of DNA methylation (GO:1905643; p(FRD) = 0.0099), TFAP2 (AP-2) family regulates transcription of cell cycle factors (R-HSA-8866911; p(FRD) = 0.0154) (Supplementary Table S3).

Fourth, rs10454142 *PPP1R21* and eight proxy loci affect the expression quantitative traits (eQTL) of 10 genes (*FOXN2*, *GTF2A1L*, *LHCGR*, *PPP1R21*, *RP11-191L17.1*, *RP11-460M2.1*, *STON1-GTF2A1L*, *FSHR*, *STON1*, and *MSH6*) in the organism, including five genes in the mammary gland (*PPP1R21*, *GTF2A1L*, *RP11-460M2.1*, *STON1-GTF2A1L*, and *MSH6*), four genes in fibroblasts (*PPP1R21*, *GTF2A1L*, *FOXN2*, *MSH6*), and one gene in the liver (*GTF2A1L*) (Table 4, Supplementary Table S4). Interestingly, the at-BC-risk allele C of this polymorphism determines the high expression of the *PPP1R21* gene in both the mammary gland and fibroblasts, but is associated with low eQTL of all other genes in the mammary gland (*GTF2A1L*, *RP11-460M2.1*, *STON1-GTF2A1L*, and *MSH6*), fibroblasts (*GTF2A1L*, *FOXN2*, and *MSH6*) and liver (*GTF2A1L*) (Supplementary Table S4).

Fifth, rs10454142 *PPP1R21* and eight LD SNPs are involved in splicing quantitative traits (sQTL) of four genes (*GTF2A1L*, *PPP1R21*, *STON1*, and *STON1-GTF2A1L*) in the organism, including three genes in the mammary gland (*GTF2A1L*, *PPP1R21*, and *STON1*) and one gene—*PPP1R21*, in fibroblasts (Table 4, Supplementary Table S5). At-BC-risk genetic variant C correlates with a low sQTL of the *PPP1R21* in both the mammary gland and fibroblasts, but it is associated with a high sQTL of the *GTF2A1L* and *STON1* genes in the mammary gland (Supplementary Table S5).

Sixth, rs10454142 *PPP1R21* and all 10 strongly linked loci affect the level of methylation of a number of genome sites in blood, immunocompetent cells (CD14+ monocytes, native CD4 + T cells), liver (carcinoma), cerebral cortex, with predominant hypermethylation effects for the polymorphic variant C rs10454142 *PPP1R21* (Supplementary Table S6). Interestingly, this is consistent with the above data, which we obtained on the directionality of the association of this allele with the expression of various genes—the allele C rs10454142 *PPP1R21*—associated mainly with hypermethylation of adjacent genome regions, determines mainly the reduced expression of the overwhelming number of genes for which it is eQTL-significant (8 genes out of 10: *FOXN2*, *GTF2A1L*, *LHCGR*, *RP11-191L17.1*, *RP11-460M2.1*, *STON1-GTF2A1L*, *FSHR*, and *MSH6*). Only in relation to two genes—*PPP1R21* and *STON1*—this allele increases transcriptional activity. And these patterns are characteristic both for the whole organism as a whole, and for the breast, fibroblasts, and liver, which have important pathophysiological significance for BC.

So, according to our data, rs10454142 *PPP1R21* and ten proxy SNPs are functionally significant in relation to eleven genes (*RP11-191L17.1*, *RP11-460M2.1*, *FSHR*, *STON1*, *STON1-GTF2A1L*, *MSH6KLRAQ1*, *FOXN2*, *GTF2A1L*, *LHCGR*, and *PPP1R21*), including eight genes in the mammary gland (*RP11-460M2.1*, *STON1-GTF2A1L*, *MSH6*, *STON1KLRAQ1*, *FOXN2*, *GTF2A1L*, and *PPP1R21*), four genes in fibroblasts (*GTF2A1L*, *PPP1R21*, *FOXN2*, and *MSH6*), and four genes in the liver (*GTF2A1L*, *PPP1R21*, *FOXN2*, and *MSH6*). The aforementioned 11 genes are involved in the processes of reproductive system development (GO:0061458; p(FRD) = 0.0040)) and interactions with hormone receptors (R-HSA-375281; p(FRD) = 0.0259) (Gene Ontology Resource data).

At the next stage of our in silico analysis, we studied the functionality of all eight SHBG-significant polymorphisms involved in predisposition to BC. It was found that three of the eight considered SNPs are functionally active in breast cell cultures—epithelial (Breast variant Human Mammary Epithelial Cells) and myoepithelial (Breast Myoepithelial Primary Cells). They were located in enhancers (rs10454142 *PPP1R21*) and active promoters (rs17496332 *PRMT6*, rs780093 *GCKR*) of these cells, respectively (Table 5). Also, five of the eight analyzed SNPs (rs780093 *GCKR*, rs10454142 *PPP1R21*, rs440837 *ZBTB10*, rs7910927 *JMJD1C*, and rs8023580 *NR2F2*) exhibit appreciable functional effects (located in the districts of enhancers, promoters, active enhancers, and active promoters) in the liver. This organ is the site of SHBG formation [18], the involvement of genetic determinants of which we study in the development of BC in this paper. The most strongly pronounced epigenetic effects in the liver have been showed by three SNPs—rs780093 *GCKR*, rs10454142 *PPP1R21*, rs440837 *ZBTB10* (Table 5)—which are located in the regions of both enhancers and promoters, including in the area of active enhancers and promoters.

**Figure 3:**
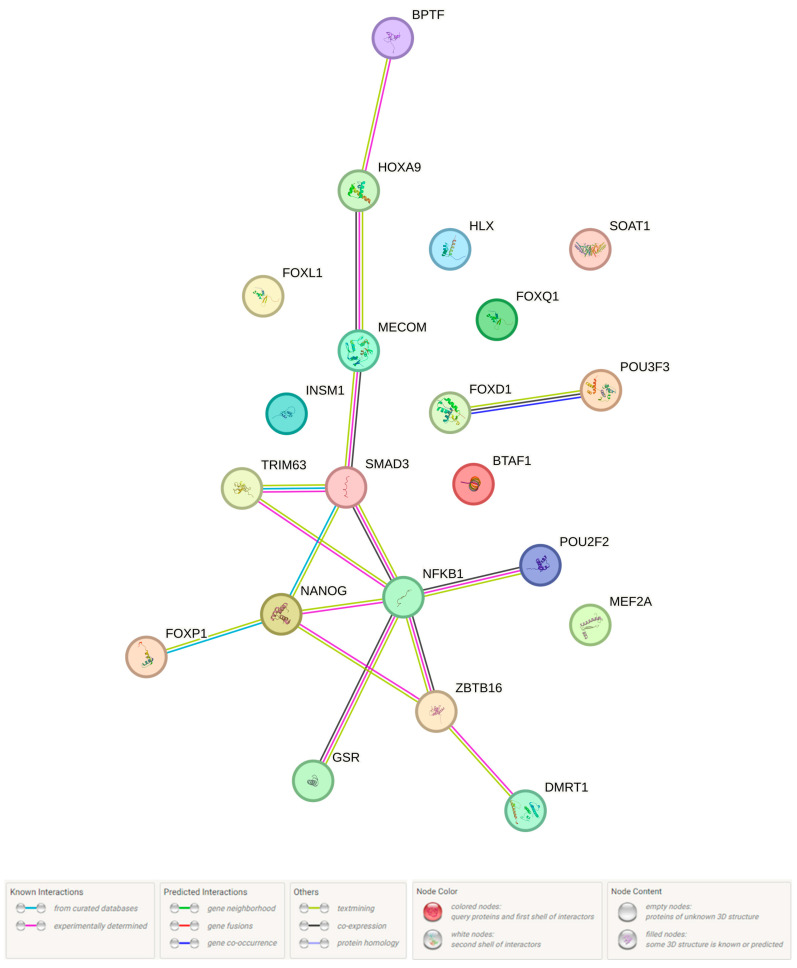
A network of transcription factors interactions linked with the breast cancer development due to eight polymorphisms of SHBG candidate genes associated with the disease (STRING data).

It was revealed that all 8 loci involved in the BC susceptibility influence the connection of regulatory DNA sites with 21 TFs (DMRT1, Evi-1, FAC1, Foxd1, Foxl1, Foxp1, Foxq1, GR, Hlx1, Hoxa9, INSM1, Mef2, Nanog, NFKB1, PLZF, Irf, Pou2f2, Pou3f3, Smad3, STAT, and TATA) (Supplementary Table S7). In obedience to the STRING database, the vast majority of these TFs (14 out of 21) interact with each other (Figure 3), and 3 TFs (NF-kappaB, Smad3, Nanog) are of fundamental importance in this case—they interact simultaneously with 4–5 other TFs. The maximum interaction effects were registered between SMAD3 and NANOG (score 0.884), SMAD3 and MECOM (score 0.858), FOXP1 and NANOG (score 0.855), NFKB1 and SMAD3 (score 0.765), and TRIM63 and SMAD3 (score 0.649). NFKB1 and SMAD3 are characterized by co-expression (co-expression score 0.135).

Interestingly, these 21 TFs are involved in many different processes (about 200!) (Supplementary Table S8) such as: (a) regulation of gene expression (activity of DNA-binding activator/repressor of transcription (GO:0001228; GO:0001227), DNA-protein complexes (GO:0032993), activity of RNA polymerase II (GO:0000122; GO:0045944), etc.); (b) morphogenesis (GO:0048729; GO:0009888; GO:0048856), including the development of endoderm (GO:0007492) and embryo (GO:0048598), etc.), development/differentiation/functioning of various organ systems (adrenal glands (GO:0030325), endocrine system (GO:0035270), kidneys (GO:0001822), muscles (GO0090257), nervous system (GO:0007399), etc.; (c) the regulation of metabolic processes (GO:0010557; GO:0031324; GO:0019219; GO:0009889), including metabolism/biosynthesis of RNA (GO:1902679; GO:0051253; GO:2001141), nitrogenous compounds (GO:0006807), the organic cyclic compounds (GO:1901362), etc.

**Figure 4:**
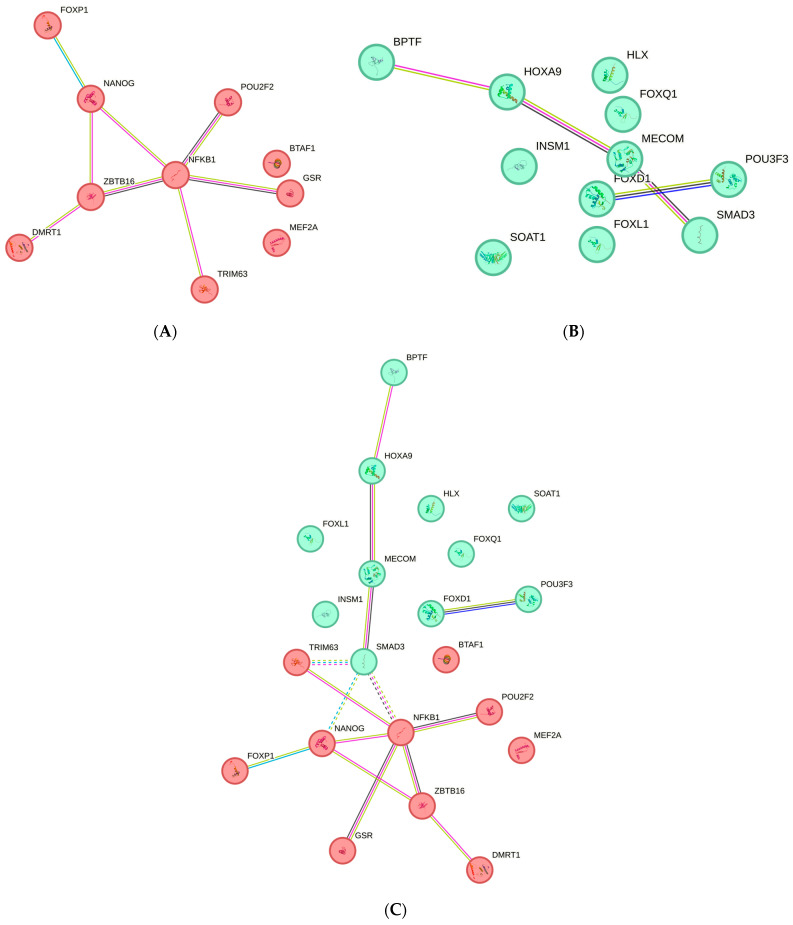
Clusters at a network of transcription factors interactions linked with the breast cancer development due to eight polymorphisms of SHBG candidate genes associated with the disease (STRING data): (**A**)—cluster 1 (indicated in red), (**B**)—cluster 2 (indicated in green), (**C**)—two clusters in total.

After performing the clustering procedure in the STRING program (used the “kmeans clustering” approach) we have identified 2 groups of clusters among the 21 TFs studied (Figure 4). The first cluster includes 10 TFs (BTAF1, DMRT1, FOXP1, GSR, MEF2A, NANOG, NFKB1, POU2F2, TRIM63, and ZBTB16) (Figure 4A) involved mainly in the processes of gene transcription regulation: the activity of the DNA-binding activator/repressor of transcription (GO:0001228; GO:0001227), the activity of RNA polymerase II (GO:0000978; GO:0000122; GO:0045944), etc. NFKB1 plays a dominating role in the 1st cluster TFs interactions, because it simultaneously interacts with five other TFs (GSR, NANOG, POU2F2, TRIM63, and ZBTB16), and also the most noticeable cooperation were registered for FOXP1 and NANOG (score 0.855), DMRT1 and ZBTB16 (score 0.599), NFKB1 and POU2F2 (score 0.561), NFKB1 and ZBTB16 (score 0.518), and NFKB1 and TRIM63 (score 0.491).

The second cluster was represented by 11 TFs (BPTF, FOXD1, FOXL1, FOXQ1, HLX, HOXA9, INSM1, MECOM, POU3F3, SMAD3, SOAT1) (Figure 4B) involved mostly in the processes of embryogenesis (GO:0048568), various organ systems morphogenesis (urogenital (GO:0001655), immune (GO:0002520), digestive (GO:0048565), adrenal glands (GO:0030325), etc.), and metabolic processes regulation (GO:0010556; GO:0031326; GO:0080090, etc.). Communications between MECOM and SMAD3 (score 0.858), HOXA9 and MECOM (score 0.456), FOXD1 and POU3F3 (score 0.432), BPTF and HOXA9 (score 0.430) are very essential for this cluster.

A significant act of all eight BC-associated loci on the DNA methylation level was revealed (Supplementary Table S9). Moreover, these connections are observed both in normal organs and cell cultures (blood, brain, uterus, immunocompetent cells (CD14 + monocytes, native CD4 + T cells)), and in tumors of various localization, such as the liver and other organs of the digestive system (pancreas, oral cavity, esophagus, stomach, thick intestines, rectum, thyroid gland, kidneys, lungs, etc.). It should be noted the most “serious” associations with the genome methylation parameters are in such SNPs as rs17496332 *PRMT6*, rs780093 *GCKR*, rs3779195 *BAIAP2L1*, and rs7910927 *JMJD1C*. It is extremely interesting that the rs17496332 *PRMT6* has been associated with the DNA methylation level (cg09367891 (chr1:107599246)) in invasive breast carcinoma (Supplementary Table S9).

At the final stage (stage 3) of our in silico study, we analyzed the summary functionality of all 130 BC-correlated loci (8 BC-causal and 122 proxy SNPs) (the Haploreg program was used). It was found that 3 SNPs (2.30%) were situated in the coding regions (exons) of genes (2 of them [rs4149056 *SLCO1B1* and rs1260326 *GCKR*] were missense substitutions and one, rs17855177 *FOXN2*, was a synonymous replacement), 1 SNP (0.77%) was placed in the 3′-UTR region of the *BRI3* gene (rs7015), and 87 SNPs (66.92%) were located in intron areas. Among the 130 analyzed loci, 10 SNPs (7.69%) were positioned in sites marking as promoters/enhancers, 37 SNPs (28.46%) were in the zones of hyper sensibility to the enzyme DNase 1, 17 SNPs (13.08%), and 115 SNPs (88.46%) were in regions of suspected binding sites with regulatory proteins and TFs, respectively (Supplementary Table S10). Overall, 124 SNPs (95.38%) have significant epigenetic potential among 130 BC-related loci (Supplementary Table S10). Among the linked loci, the most expressed regulatory potential was revealed for the rs10761751 and rs10761758 loci of the *JMJD1C* gene (strongly linked to the BC-causal locus rs7910927 *JMJD1C*; according to our data, it is part of five models of intergenic interactions that are risky for BC); these loci affect the DNA communication with 27 and 24 TFs, respectively (Supplementary Table S10). So, 8 BC-associated loci and 116 strongly linked SNPs are functionality in relation to 14 genes, such as *PRMT6*, *BAIAP2L1*, *BRI3*, *GCKR*, *JMJD1C*, *KLRAQ1*, *NR2F2*, *PPP1R21*, *SLCO1B1*, *RP11-327J17.2*, *RP11-327J17.3*, *FOXN2*, *RP11-48B3.4*, and *ZBTB10* (Supplementary Table S10).

One BC-causal locus (rs4149056 *SLCO1B1*) and one locus linked to another BC-causal locus (rs1260326 *GCCR* in LD [r^2^ = 0.91; D’ = 0.96] with BC-associated rs780093 *GCCR*) lead to amino acid substitutions (Val174Ala SLCO1B1 and Leu446Pro GCCR, respectively) having the predictive class “deleterious (SIFTscore-0.002)/probably damaging(Polyphen2score-1.000)” and “tolerated (SIFTscore-0.747)/possibly damaging (Polyphen2score-0.806)”, respectively.

We found serious eQTL correlations of 7 BC-associated loci (with the exception of rs440837 *ZBTB10*) and 97 strongly linked SNPs (97/122, 79.51%) with 41 genes such as *AC004967.7*, *AC074117.10*, *ASNS*, *ATRAID*, *BAIAP2L1*, *BRI3*, *C2orf16*, *FNDC4*, *FOXN2*, *FSHR*, *GCKR*, *IFT172*, *GTF2A1L*, *GPN1*, *JMJD1C*, *JMJD1C-AS1*, *KRTCAP3*, *LHCGR*, *LMTK2*, *MSH6*, *MRPL35P2*, *NRBF2*, *NRBP1*, *PPM1G*, *PPP1R21*, *PRMT6*, *REEP3*, *RPL7AP50*, *RP11-191L17.1*, *RP11-307C18.1*, *RP11-327J17.2*, *RP11-460M2.1*, *SLC5A6*, *SLCO1B3*, *SLCO1B7*, *SNX17*, *STON1*, *STON1-GTF2A1L*, *TECPR1*, *TRIM54*, and *ZNF512* in multiple organs (Supplementary Tables S11 and S12), including the mammary gland [BC target organ] (**10 genes**): *ATRAID*, *GTF2A1L*, *MRPL35P2*, *MSH6*, *NRBP1*, *PPP1R21*, *PRMT6*, *RP11-307C18.1*, *STON1-GTF2A1L*, and *RP11-460M2.1*; fibroblasts: *ATRAID*, *AC074117.10*, *BAIAP2L1*, *BRI3*, *FOXN2*, *MRPL35P2*, *JMJD1C-AS1*, *RP11-307C18.1*, *NRBF2*, *JMJD1C*, *GTF2A1L*, *GPN1*, *MSH6*, *NRBP1*, *PPP1R21*, *PRMT6*, and *SLC5A6*; liver (*BRI3*, *GTF2A1L*, *PRMT6*, *RP11-307C18.1*, and *RP11-327J17.2*), visceral (*AC074117.10*, *GTF2A1L*, *KRTCAP3*, *MRPL35P2*, *NRBP1*, *PRMT6*, *REEP3*, *RP11-307C18.1*, *BRI3*, *RP11-460M2.1*, and *STON1-GTF2A1L*), and subcutaneous (*ATRAID*, *BRI3*, *GTF2A1L*, *MRPL35P2*, *NRBP1*, *PPM1G*, *PPP1R21*, *PRMT6*, *RP11-307C18.1*, and *STON1-GTF2A1L*) adipose, pituitary (*FOXN2*, *GTF2A1L*, *MRPL35P2*, and *RP11-307C18.1*), and hypothalamus (*PPP1R21*, *PRMT6*, and *RP11-307C18.1*), ovaries (*RP11-307C18.1*), thyroid (*AC074117.10*, *ATRAID*, *BAIAP2L1*, *C2orf16*, *FNDC4*, *GCKR*, *GTF2A1L*, *IFT172*, *JMJD1C-AS1*, *KRTCAP3*, *LMTK2*, *MRPL35P2*, *PPM1G*, *PPP1R21*, *PRMT6*, *REEP3*, *RP11-307C18.1*, *RPL7AP50*, *STON1*, *TECPR1*, and *ZNF512*), adrenal glands (*FOXN2*, *GTF2A1L*, *KRTCAP3*, *MRPL35P2*, *PRMT6*, and *RP11-307C18.1*), muscles (*ASNS*, *BAIAP2L1*, *BRI3*, *GPN1*, *GTF2A1L*, *KRTCAP3*, *MRPL35P2*, *PPM1G*, *PPP1R21*, *PRMT6*, *RP11-307C18.1*, and *SNX17*), blood (*KRTCAP3*, *NRBP1*, *PRMT6*, *TECPR1*, and *RP11-307C18.1*), involved in the disorder pathophysiology.

Importantly, among the eQTL-significant 7 BC-associated loci and 97 proxy SNPs, those correlated with mRNA production in the mammary gland were 3 SNPs (rs10454142 *PPP1R21*, rs7910927 *JMJD1C*, and rs3779195 *BAIAP2L1*) (3 out of 8 studied loci, 37.50%) and 84 LD SNPs (84 out of 122 studied loci, 68.85%); fibroblasts—5 variants (rs17496332 *PRMT6*, rs780093 *GCKR*, rs10454142 *PPP1R21*, rs7910927 *JMJD1C*, and rs3779195 *BAIAP2L1*) (5/8, 62.50%), and 93 LD SNPs (93/122, 76.23%); liver—4 loci (rs17496332 *PRMT6*, rs10454142 *PPP1R21*, rs3779195 *BAIAP2L1*, and rs8023580 *NR2F2*) (4/8, 50.00%) and 37 proxy SNPs (37/122, 30.33%) (Supplementary Tables S11 and S12).

We discovered a connection of 3 BC-causal loci (rs780093 *GCKR*, rs10454142 *PPP1R21*, and rs3779195 *BAIAP2L1*) and 31 strongly linked SNPs (31/122, 25.41%) with sQTL of 13 genes (*BAIAP2L1*, *BRI3*, *FNDC4*, *GCKR*, *GPN1*, *GTF2A1L*, *IFT172*, *KRTCAP3*, *PPP1R21*, *SNX17*, *STON1*, *STON1-GTF2A1L*, and *TRIM54*) in various organs (Supplementary Tables S13 and S14), including the mammary gland (*IFT172*, *FNDC4*, *PPP1R21*, *GTF2A1L*, *STON1*, and *BRI3*), fibroblasts (*FNDC4* and *PPP1R21*), liver (*GCKR* and *FNDC4*), visceral (*IFT172*, *FNDC4*, *GTF2A1L*, *STON1*, *PPP1R21*, *BRI3*, and *STON1-GTF2A1L*), and subcutaneous (*IFT172*, *FNDC4*, *GTF2A1L*, *STON1*, *PPP1R21*, and *BRI3*) adipose, pituitary (*IFT172* and *PPP1R21*), ovaries (*IFT172*), thyroid (*IFT172*, *KRTCAP3*, *PPP1R21*, and *BRI3*), adrenal glands (*IFT172* and *FNDC4*), muscles (*IFT172*, *TRIM54*, and *BRI3*), brain (hypothalamus, cortex, basal ganglia, etc.) (*FNDC4* and *BRI3*), which are important for BC biology.

In total, 8 BC-causal polymorphisms and 122 proxy SNPs due to their epigenetic effects (14 genes: *PRMT6*, *BAIAP2L1*, *BRI3*, *GCKR*, *JMJD1C*, *KLRAQ1*, *NR2F2*, *PPP1R21*, *RP11-327J17.2*, *SLCO1B1*, *ZBTB10*, *RP11-327J17.3*, *FOXN2*, and *RP11-48B3.4*), missense substitutions (2 genes: *SLCO1B1* and *GCKR*), acts on gene expression (41 genes: *AC004967.7*, *AC074117.10*, *ASNS*, *ATRAID*, *BAIAP2L1*, *BRI3*, *C2orf16*, *FNDC4*, *FOXN2*, *FSHR*, *GCKR*, *IFT172*, *GTF2A1L*, *GPN1*, *JMJD1C*, *JMJD1C-AS1*, *KRTCAP3*, *LHCGR*, *LMTK2*, *MSH6*, *MRPL35P2*, *NRBF2*, *NRBP1*, *PPM1G*, *PPP1R21*, *PRMT6*, *REEP3*, *RP11-191L17.1*, *RP11-307C18.1*, *RP11-327J17.2*, *SLC5A6*, *RPL7AP50*, *RP11-460M2.1*, *SLCO1B3*, *SLCO1B7*, *SNX17*, *STON1*, *STON1-GTF2A1L*, *TECPR1*, *TRIM54*, and *ZNF512*), and splicing (13 genes: *BAIAP2L1*, *FNDC4*, *GCKR*, *IFT172*, *GPN1*, *GTF2A1L*, *KRTCAP3*, *PPP1R21*, *BRI3*, *SNX17*, *STON1*, *STON1-GTF2A1L*, and *TRIM54*) are functionality in respect of 48 genes (*PRMT6*, *AC004967.7*, *AC074117.10*, *ASNS*, *ATRAID*, *BAIAP2L1*, *BRI3*, *C2orf16*, *FNDC4*, *FOXN2*, *FSHR*, *GCKR*, *GTF2A1L*, *IFT172*, *JMJD1C*, *JMJD1C-AS1*, *LHCGR*, *GPN1*, *KRTCAP3*, *KLRAQ1*, *LMTK2*, *MRPL35P2*, *MSH6*, *NR2F2*, *NRBF2*, *PPP1R21*, *PPM1G*, *RP11-191L17.1*, *NRBP1*, *PRMT6*, *REEP3*, *RP11-307C18.1*, *RP11-327J17.2*, *RP11-327J17.3*, *RP11-460M2.1*, *RP11-48B3.4*, *RPL7AP50*, *SLC5A6*, *SLCO1B1*, *SLCO1B3*, *STON1-GTF2A1L*, *SNX17*, *SLCO1B7*, *STON1*, *TECPR1*, *TRIM54*, *ZBTB10*, and *ZNF512*).

**Figure 5:**
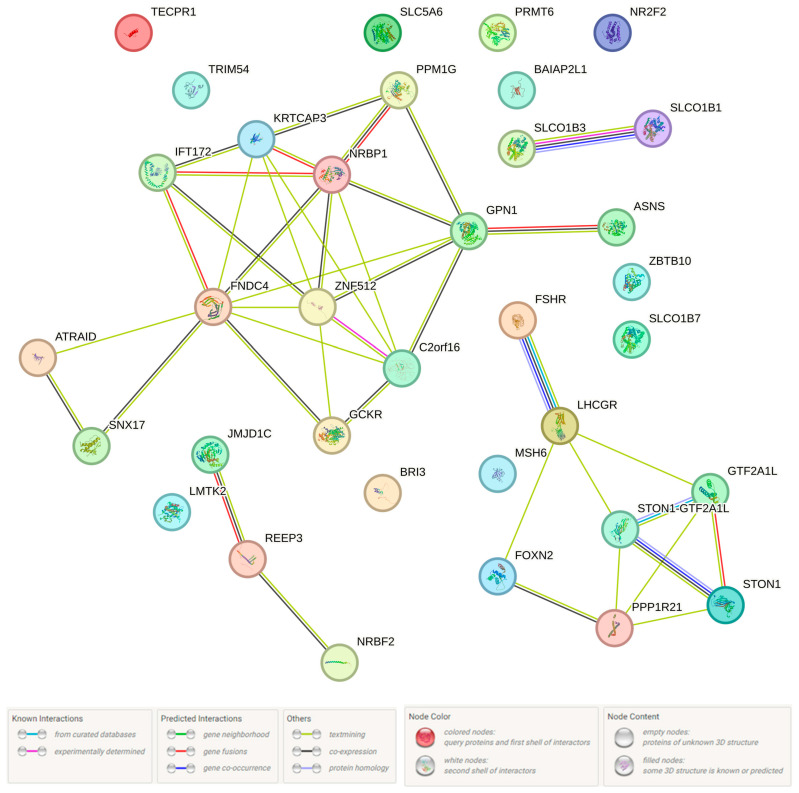
A network of protein interactions involved in the breast cancer development due to candidate genes functionally associated with 8 BC-associated loci and strongly linked to them by 122 SNPs (STRING data).

Using the STRING program, protein interactions controlled by the above 48 genes were studied (Figure 5). These interactions were carried out with the participation of organic anion transporter polypeptide (SLCO1B1, SLCO1B3, and SLCO1B7) (IPR004156; p(FRD) = 0.0202)) (InterPro data), transcription factor IIA, alpha/beta subunit (STON1-GTF2A1L and GTF2A1L) (SM01371; p(FRD) = 0.0150) and Kazal type serine protease inhibitors (SLCO1B1, SLCO1B3, and SLCO1B7) (SM00280; p(FRD) = 0.0388) (SMART data). The most prominent interworking was found for proteins of the following genes: *STON1* and *GTF2A1L* (score 0.992), *REEP3* and *JMJD1C* (score 0.901), *ZNF512* and *C2orf16* (score 0.777), *LHCGR* and *FSHR* (score 0.698), *NRBP1* and *KRTCAP3* (score 0.626), *STON1* and *PPP1R21* (score 0.610), *GPN1* and *C2orf16* (score 0.610), and *ZNF512* and *KRTCAP3* (score 0.604). Apart from this, co-expression was revealed in the transcriptional activity of a number of the genes under consideration: *SLCO1B3* and *SLCO1B1* (score 0.267), *SNX17* and *ATRAID* (score 0.151), *PPM1G* and *GPN1* (score 0.146), *NRBP1* and *GPN1* (score 0.138), *GCKR* and *FNDC4* (score 0.127), and *FSHR* and *LHCGR* (score 0.124). In pursuant to Gene Ontology Resource data, BC candidate genes/proteins influence on the sodium-independent organic anion transmembrane transporter activity (GO:0015347; p(FRD) = 0.0235)) and the secondary active transmembrane transporter activity (GO:0015291; p(FRD) = 0.0414) (data from GO molecular function complete and PANTHER GO-Slim Molecular Function, respectively).

At the end of study, using the regBase-CG database, we in silico assessed the potential role of the eight BC-involved polymorphisms of SHBG candidate genes as drivers of tumor development. The results obtained are presented in Table 6. It was found that two SNPs out of eight considered loci, rs10454142 *PPP1R21* and rs4149056 *SLCO1B1*, are the likely drivers of the occurrence of tumors (“likely cancer driver”) (Table 6). Importantly, according to our data, rs10454142 *PPP1R21* was both an independent risk factor for BC (OR = 1.31) and part of all nine significant BC-associated models, and rs4149056 *SLCO1B1* was associated with BC within three models of intergenic interactions.

### 2.2. The Final Results Assessing the Functionality of BC-Related Loci

So, summarizing the materials obtained in this section of the work, devoted to the assessment of the functionality of BC-associated loci and strongly linked polymorphisms, we can note the following: Firstly, the presence of pronounced functional effects (epigenetic modifications, eQTL, and sQTL) of BC-risky SNP rs10454142 *PPP1R21* and 10 proxy SNPs in relation to 11 genes, including BC biology, important to organs/cells such as the mammary gland (8 genes), fibroblasts (4 genes), and liver (4 genes), were found. Secondly, it was revealed that all 8 loci determining the predisposition to BC influence the connection of regulatory DNA sites, with 21 TFs which can potentially be involved in many different BC-significant processes (about 200), such as the regulation of gene expression, morphogenesis, and the development/differentiation/functioning of various organ systems, regulating metabolic processes. Thirdly, 130 BC-correlated loci (8 BC-causal and 122 proxy SNPs) exhibit their functionality with respect to 48 different genes in the interaction of protein products, of which polypeptides-organic anion carriers, IPA transcription factors, alpha/beta subunits, and serine protease inhibitors of the Kazal type were important.

## 3. Discussion

In this report, we showed the risk value for BC of SHBG-lowering allele variant C rs10454142 *PPP1R21* (OR = 1.31) in Caucasian women in Russia, which determines 0.32% of the cancer variance. Also, eight of the nine studied SHBG-related GWAS-impacted SNPs have been involved in BC susceptibility as part of nine gene interaction models, the greatest contribution to which was made by rs10454142 *PPP1R21* (included in all nine models, 100%) and some other loci (rs7910927 *JMJD1C*, rs17496332 *PRMT6*, rs780093 *GCKR*, and rs440837 *ZBTB10*). For SHBG-related loci, pronounced functionality in the organism (including breast, liver, fibroblasts, etc.) was predicted in silico, having a direct relationship through many pathways with cancer pathophysiology.

In GWAS results of 7046 individuals performed by Coviello et al. in 2012, the association of rs10454142 *PPP1R21* (2p16.3) with the level of circulating SHBG in the organism was detected, and the “major” variant T of this polymorphism was linked with a higher content of this protein (β = 0.026) [23]. The GWAS materials indicate associations of several loci strongly linked to rs10454142 *PPP1R21* with the various liver enzymes level: alanine aminotransferase (rs10208627, *p* = 3 × 10^−9^ [34]; r^2^ = 0.56, D′ = 1.00), alkaline phosphatase (rs6749773, *p* = 1 × 10^−13^ [34], *p* = 4 × 10^−9^ [35], *p* = 1 × 10^−15^ [36]; r^2^ = 0.53, D′ = 1.00), and gamma-glutamyltranspeptidase (rs13429377, *p* = 3 × 10^−22^ [34]; r^2^ = 0.59, D′ = 1.00). It should be noted that the liver is the site of SHBG synthesis [18] and, at the same time, as shown by our in silico data, rs10454142 *PPP1R21* and strongly linked loci are functionally active in liver cells (localized in regions of histone proteins marking enhancers/promoters/active enhancers/active promoters associated with the *GTF2A1L* gene expression and correlated with the methylation of a number of genome sites in liver carcinoma).

Importantly, the allele C rs10454142 *PPP1R21*, which is risky for BC (our data), correlates with a low concentration of circulating SHBG in the organism (GWAS data Coviello et al. [23]). Using the MR method (this method allows to evaluate the cause-effect relationships between the studied features [37]), the relationship of SNPs and GWAS-associated with the level of SHBG [23] with the BC risk in postmenopausal women was shown; furthermore, an increase in the concentration of circulating SHBG (for every 25 nmol/L) led to a decrease in the risk of BC as a whole (OR = 0.94, *p* = 0.006) and ER-positive cancer (OR = 0.92, *p* = 0.003), but the risk of developing ER-negative tumors, on the contrary, increased (OR = 1.09, *p* = 0.047) [24]. Similar data (reduction of the risk of ER-positive BC and an increase in the risk of ER-negative BC with an increase in SHBG levels) were obtained in the study of Chen et al., performed on the basis of the MR of Breast Cancer Association Consortium data [14]. So, our results on the BC risk role of the SHBG-lowering GWAS-significant allele C rs10454142 *PPP1R21* are completely consistent with the foregoing literature data, according to which SHBG-increasing alleles of GWAS-significant SNPs correlate with a low risk of BC (in general and in ER-positive). Importantly, in the sample of patients studied by us, the majority of patients had ER-positive BC (66%) and postmenopausal status (68.16%).

We have shown that eight out of nine studied polymorphisms affecting the concentration of SHBG in the organism according to GWAS data (rs17496332 *PRMT6*, rs780093 *GCKR*, rs10454142 *PPP1R21*, rs3779195 *BAIAP2L1*, rs440837 *ZBTB10*, rs7910927 *JMJD1C*, rs4149056 *SLCO1B1*, and rs8023580 *NR2F2* [23]) interact between by themselves and determine the predisposition to BC. Previously performed genetic studies have shown that the contribution of hereditary factors in determining the level of circulating SHBG in women reaches 56–58% [17,22], and, at the same time, the GWAS-significant polymorphisms studied in this work have a significant role in the genetic variability of SHBG concentration in women (~8.4%) [23]. Importantly, in the earlier work, Dimou et al., using the MR method, showed the relationship of the same SNP list (GWAS-associated with the level of SHBG [23]) with the risk of BC in postmenopausal women [24]. It is very interesting that two polymorphisms (rs10454142 *PPP1R21* and rs4149056 *SLCO1B1*) out of eight BC-associated loci are likely drivers of the occurrence of tumors (“likely cancer driver”) (prognostic estimates were obtained by us using the regBase-CAN database).

SHBG is a glycoprotein (90–100 kDa) consisting of two identical peptide chains, each of which contains steroid-binding sites [18]. SHBG is produced mainly in the liver (by hepatocytes), but there is evidence of its formation in the mammary gland, brain, uterus, ovaries, placenta, etc. [38]. It has been shown that thyroid hormones and estrogens increase the production of SHBG, and that pro-inflammatory cytokines, on the contrary, reduce the formation of SHBG (due to the regulation of the expression of hepatocyte nuclear factor 4 alpha) [18]. Due to the presence of steroid-binding sites, SHBG binds and transports testosterone, estradiol, and other sex steroids in plasma, thus affecting their bioavailability [18]. SHBG is characterized by the following sequence of “preferences” in the binding of sex hormones (in descending order of affinity): dihydrotestosterone > testosterone > androstenediol > estradiol > estrone. Thus, SHBG has a higher affinity for testosterone and a lower affinity for estradiol [39].

There are inverse correlations between the concentration of circulating SHBG and the level of bioavailable (active) testosterone and estrogens in a woman’s organism [38,40]. Importantly, the concentration of free (active) testosterone depends very much on the concentration of SHBG in plasma, since only 1–2% of testosterone in circulating blood is free (unbound) and therefore active, whereas 65% of testosterone is associated with SHBG, and the rest of its amount (>30%) is associated with albumin [39]. The following data are available for estradiol: 38% associated with SHBG, 60% with albumin, and only 2% are bioavailable (active) [19]. Thus, women with low levels of SHBG will have an increased level of free (active) testosterone and estrogens, and vice versa, a high content of circulating SHBG will cause low concentrations of bioavailable testosterone and estrogens in the organism.

There is convincing evidence of a negative genetic relationship between the levels of SHBG and free testosterone in women [17,20]. Based on the analysis of family data of 2685 women from the Framingham Heart Study (868 pairs of sisters and 688 pairs of mother-daughter were studied) by Coviello et al., negative genetic correlations were established between SHBG and free testosterone (p_G_ = −0.60) and direct genetic correlations between SHBG and total testosterone (p_G_ = 0.31) [17]. Sinnott-Armstrong et al. (a GWAS analysis of UK Biobank data was made) found strong reverse genetic correlations between SHBG and bioavailable testosterone in women (r_g_ = −0.75), while no reliable links were found between SHBG and total testosterone in women (r_g_ = −0.035) [20].

Numerous data from the literature specify an important pathogenetic role of SHBG in BC [13,14,21,24,41,42,43,44,45]. It has been convincingly shown that the increased content of circulating SHBG has a protective value for the development of the disease; however, these relationships (their presence and orientation) may depend on the pre/postmenopausal status of women and the molecular subtype of the tumor [13,14,24,44,45]. In the meta-analysis performed by Drummond et al., they showed a dose-dependent association of high levels of SHBG with a low risk of developing BC in postmenopausal women (OR = 0.54) and the absence of significant relationships in premenopausal women (OR = 0.96; *p* > 0.05) and in groups of patients with different ER status (positive/negative) [45]. Similar data were obtained in the work of Arthur et al. for ductal carcinoma in situ: the level of SHBG had inverse correlations with the risk of disease in postmenopausal women (HR = 0.75) and was not associated with the disorder in premenopausal women [44]. Slightly different results were obtained in the study of Tin Tin et al., in which the unidirectional effect of SHBG (the protective value of an increased level of this protein in BC) was established in both premenopausal (OR = 0.96) and postmenopausal (OR = 0.89) women [13]. In Chen et al., reverse associations of SHBG with ER-positive tumors (OR = 0.83) and direct associations with ER-negative (OR = 1.12) and triple negative tumors (OR = 1.19) were shown [14]. Similarly, Dimou et al. demonstrated a protective value of high SHBG content in BC in general (OR = 0.94) and in ER-positive tumors (OR = 0.92), and a risky value in ER-negative cancer (OR = 1.09) [24].

The correlation of SHBG with BC risk may be based on the following mechanisms. Firstly, SHBG is of paramount importance in regulating the level of bioavailable (active) testosterone and estrogens in the female [18,19,38,39,40,42], and due to this (the modulation of the phenotypic effects of testosterone and estrogens), it can be involved in the BC pathophysiology (these mechanisms will be discussed in detail below) [39,42,43,45]. Herewith, the high content of circulating SHBG will cause its “maximum” binding to testosterone and estrogens, and lead to low levels of bioavailable (active) sex hormones in the organisms of women, which will eventually manifest themselves in the minimally pronounced phenotypic effects of testosterone and estrogens [38,40].

Secondly, SHBG can independently potentiate various intracellular BC-important effects (increase in intracellular cAMP, activation of protein kinase A, inhibition of MAP kinase pathways, etc.), due to binding to specific, high affinity membrane receptors in various human tissues (hypothalamus, endometrium, placenta, etc.) [43]. It is important that only SHBG unrelated to sex hormones can interact with membrane receptors, and, at the same time, if sex steroids initially bind to SHBG, they prevent its interaction with cellular receptors and, accordingly, block its intracellular effects [43]. There are experimental data on the interaction of the “SHBG-membrane” in estrogen-dependent BC cells MCF-7 [43]. The end result of the intracellular effects of SHBG is a decrease in the proliferative activity of cells and the induction of apoptosis, which is of protective importance in BC development [42].

Thirdly, SHBG can inhibit the action of estrogens in BC cells [43]. This effect can be achieved in two ways: (a) It is believed that after binding to the membrane receptor, SHBG can again bind steroids with the same affinity as in solution [43]. Thus, the additional “anti-estrogen” effect of SHBG will manifest itself if the “correct” sequence of its binding, first with the cell membrane (leads to the implementation of a cascade of intracellular anti-proliferative processes), and then with free steroids, which will lead to a decrease in the number of their bioavailable forms and, accordingly, to less pronounced independent phenotypic effects in the organism (reducing the BC risk). (b) SHBG can modulate the activity of estrogen-dependent genes involved in the processes of cell growth and apoptosis, thus leading to the inhibition of genes suppressing apoptosis (*bcl-2*, *c-myc*, *EGF-R*, *PR*, etc.), ultimately causing the restoration of apoptosis in BC cells [43]. Thus, ultimately, the action of SHBG is aimed at inhibiting estrogen-mediated cell proliferation and anti-apoptosis.

According to the available literature data, SHBG is of paramount importance in determining the level of “active” (bioavailable) testosterone in the female [17,20,39], whose role in the development of BC has been proven in large-scale epidemiological studies [13,15]. In the Tin Tin et al. study, based on the exploration of serum testosterone levels levels in 30565 premenopausal (527 of them had BC) and 133294 postmenopausal women (2997 of them had BC) showed a risk value of both total (HR = 1.18; 95%CI: 1.14–1.23) and free (HR = 1.31; 95%CI: 1.23–1.40) testosterone for BC in postmenopausal women and the absence of its association with the disease in premenopausal women [13]. In the large-scale work of Tang et al., performed using MR of genetic data of the large sample of women (n = 420, 000) from UK Biobank (n = 194, 174) and the Breast Cancer Association Consortium (n = 228, 951), they demonstrated a direct genetic link between BC risk and testosterone levels (OR = 1.12) [15].

The mechanisms underlying the association of testosterone with BC are poorly understood and remain largely unclear [13,44]. The literature provides the following pathophysiological justifications for the association of testosterone with BC, while, as a rule, a higher testosterone content has a risk value for BC [13,44,46,47,48,49]. Firstly, testosterone under the action of aromatase can be converted into estradiol in adipose tissue and other organs, including breast tumor cells, and thus realize its BC risk effects through estrogen-mediated pathophysiological mechanisms that are risky for BC [13].

Secondly, testosterone participates in the control of the mammary epithelium growth due to a balanced interaction between its two active metabolites—estradiol (stimulates cell proliferation) and dihydrotestosterone (inhibits cell proliferation)—and, at the same time, an increased content of testosterone in the organism leads to higher production of estrogens and, accordingly, to hyperproliferation of cells, which is not balanced by antiproliferative action dihydrotestosterone [46]. It is assumed that this shift in the balance of androgens and estrogens lies in the genesis of ER-positive tumors [46].

Thirdly, testosterone can interact directly with androgen receptors that are present in breast tumor cells [47]. Androgen receptors are located in the cytoplasm and, in the absence of ligands (androgens), bind to heat shock proteins [50]. As soon as androgens enter the cell, they connect with their receptors, while this complex (androgens–androgen receptors) detaches from heat shock proteins and transfers to the nucleus, where, interacting with various co-stimulators, co-repressors, and transcription regulators (miR-204, SOX-4, FOXA1, etc.), modulates the expression of a number of genes (*HER3*, *MYC*, *PTEN*, *GPER*, etc.) associated with the apoptosis, differentiation, angiogenesis, and proliferation of cells, including tumor cells (Wnt/β-catenin signaling pathway, PI3K/AKT, etc.) [48,49].

Fourth, the mammary gland is a modified apocrine gland, which (its apocrine cells), under the stimulating action of androgens, synthesizes the epidermal growth factor; the interaction of this growth factor with the corresponding receptors (receptors of epidermal growth factor and human epithelial growth factor 2) leads to the “activation” of cell proliferation [46].

The literature materials indicate a significant effect of SHBG on the content of “active” (bioavailable) estradiol in the female [19]. Herewith, the role of estrogens in the occurrence of BC (and primarily ER-positive BC) has been confirmed in multitudinous studies [21,41,42,43,44,51,52,53]. The literature indicates several biological mechanisms underlying the risk effect of estrogens on BC development [44,52,53].

Firstly, it is believed that estrogens increase the proliferative activity of breast epithelial cells, and, at the same time, during more frequent DNA reduplications of these cells, the probability of mutations increases, which can potentiate the subsequent tumor transformation of breast epithelial cells [52,53].

Secondly, increased proliferation of breast epithelial cells under the action of estrogens is accompanied by the increased functional activity of mitochondria (provide additional energy synthesis for excessively proliferating cells), which can potentially lead to an increase in the content of reactive oxygen forms (have a damaging effect on DNA) as a byproduct of mitochondrial oxidative phosphorylation processes, thereby contributing to tumorogenesis in the mammary gland [51,52,53].

Thirdly, estrogen metabolites (semiquinones and quinones) have mutagenic properties, and can lead to DNA damage due to the formation of adducts and reactive oxygen forms [54,55]. Moreover, estrogen metabolites interacting directly with DNA do not require the presence of estrogen receptors to realize their pathogenic effects, which may explain the connection of estrogens with the development of ER-negative BC [53,56].

Fourth, estrogens can cause disturbances in cellular responses to DNA damage (kinase mechanisms of cell assessment of the scale and severity of DNA damage are disrupted in order to initiate cell cycle arrest, repair, or, in the case of irreparable damage, apoptosis), and DNA repair mechanisms (excision repair, nucleotide excision repair, and mismatch repair) [52,53].

Estrogens, as rules, realize their pathogenic effects in BC by interacting with their receptors (ER) through genomic (regulation of genes expression responsible for growth, differentiation, apoptosis, and angiogenesis) and non-genomic (interaction with various proteins, including adaptive proteins, G-proteins, growth factor receptors (EGFR, IGFR1, and HER2), cytoplasmic kinases (MAPKs, PI3K, and AKT), signaling enzymes (adenyl cyclase), etc.) and other mechanisms [53,57]. Thus, estrogens, having pronounced mitogenic and mutagenic effects, and stimulate proliferation and carcinogenesis in 60–70% of BC cases [51]. It is believed that an increased level of estrogen in the blood serum leads to an increase in the risk of developing BC by 2–2.5 times [58].

Thus, a decrease in the level of SHBG in the organism (determined by SHBG-lowering genetic determinants) leads to an increase in the concentration of bioavailable (“free”) testosterone and estrogens and, accordingly, causes an increase in their pro-oncogenic phenotypic effects and a decrease in the independent anti-oncogenic effects of SHBG, which ultimately has a risky value for BC development. The above pathophysiological justification may be the basis for the association of the SHBG-lowering allele variant T rs10454142 *PPP1R21* with an increased risk of developing BC.

The correlations of rs10454142 *PPP1R21* with BC, established in our study, can be determined not only with the effect of this polymorphism on the SHBG level and, accordingly, on the concentration of SHBG-dependent sex hormones (testosterone, estrogens) in the organism (we discussed these mechanisms in detail above), but may also be defined by the functional effect of this polymorphism on the epigenetic modifications, expression, and splicing of other genes (data obtained by us in silico) significant for BC pathophysiology, such as *PPP1R21*, *STON1-GTF2A1L*, *STON1*, *GTF2A1L*, *KLRAQ1*, *FOXN2*, *LHCGR*, *RP11-191L17.1*, *RP11-460M2.1*, *MSH6*, and *FSHR.*

For example, the *PPP1R21* gene (located in 2p16.3; the region of this gene is the BC-associated rs10454142, which, according to our in silico data, affects the epigenetic modifications, expression, and splicing of this gene) encodes the protein—regulatory subunit 1 of protein phosphatase 21—which belongs to the group widely represented in the organism and quite numerous in phosphoprotein phosphatase 1 (PPP1) (more than 200 PPP1 have been identified to date) [59]. PPP1s can act as target subunits, substrates, and regulators of activity in the process of reversible phosphorylation of various proteins (dephosphorylation stage) involved in intracellular signaling mechanisms in various signaling pathways “involved” in the regulation of cell growth, cell cycle, apoptosis, and other cancer-significant cellular reactions [59]. Along with this, it is assumed that PPP1R21 plays an important role in the functioning of endosomes (lysosomes), including in the sorting and maturation of endosomes, which is of paramount importance in ensuring the effective operation of the intracellular endosomal–autophagic–lysosomal system [60].

Multitudinous previously conducted clinical and experimental studies have shown that the association of *PPP1R21* (PPP1R21) with the development of various tumors, including such as colorectal carcinoma [61,62], oral cancer (in mice) [63], thyroid carcinoma [64], lung cancer [65,66], small intestine tumor [67], and stomach cancer [68]. Attention is drawn to the presence of evidence of the involvement of *PPP1R21* in oncogenesis (colorectal cancer) in genome-wide studies [62].

In several studies, the correlation of *PPP1R21* with BC has been demonstrated [69,70]. According to the materials of Cebrià-Costa et al., the expression of the *PPP1R21* gene increases in cell lines with the *LOXL2* gene “turned off” in the TNBC form of BC [69]. On the contrary, in the work of Horvath et al., a very low density of “expressed” SNPs was demonstrated in the chr2 region:48000000-48999999 containing the genes *PPP1R21*, *MSH6*, *FBX011*, *FOXN2*, *STON1*, *GTF2A1L*, and *LHCGR* in patients with all three analyzed BC subtypes (TNBC, non-TNBC, and HER2 positive) [70]. There is evidence of an association of increased expression of another representative of phosphoprotein phosphatase 1—PPP1R14C, with an increased risk of development and a poor prognosis (metastasis) with the TNBC variant of BC [71].

Interestingly, polymorphisms localized in the region of the *PPP1R21/FOXN2/PPP1R21-DT* genes are associated at the GWAS confidence level (*p* ≤ 5 × 10^−8^) with such BC-significant signs as the concentration of circulating SHBG (rs200883214 [72], rs4497915 [73], and rs11690748 [72,74]), anthropometric indicators (growth (rs4953579, rs7566996 [75], and rs76154567 [34,76])), waist circumference (rs72820455 [77]), body fat content (rs4497915 [73]), lipid profile indicators (triglycerides, high and low density lipoproteins (rs4497915 [73]), and high density lipoproteins (rs12713007 [78])), and DNA methylation (rs192224341 [79]). There are GWAS data on the relationship of the SNP of this genome region with the liver enzyme level in blood serum, such as alanine aminotransferase and aspartate aminotransferase (rs4497915 [73]), alanine aminotransferase (rs10208627 [34] and rs4290706 [36]), gamma-glutamyltranspeptidase (rs13429377 [34]), gamma-glutamyltransferase (rs62137009 [36]), and alkaline phosphatase (rs6749773 [34,35,36]). Herewith, importantly, the liver is the main place of SHBG formation in the organism [37], and the liver state, assessed by the level of liver enzymes, will directly correlate with its SHBG production.

At the end of the discussion of the obtained materials, we consider it important to note that our work revealed significant regulatory effects of SHBG-significant BC-associated loci in fibroblasts in relation to 18 genes (eQTL [17 genes]: *PPP1R21*, *ATRAID*, *AC074117.10*, *BRI3*, *BAIAP2L1*, *FOXN2*, *GPN1*, *MRPL35P2*, *JMJD1C-AS1*, *RP11-307C18.1*, *NRBF2*, *NRBP1*, *MSH6*, *JMJD1C*, *GTF2A1L*, *PRMT6*, and *SLC5A6*; sQTL [2 genes]: *FNDC4* and *PPP1R21*). The materials presented in the literature indicate the important role of fibroblasts in the tumor process, including in BC [80,81]. Fibroblasts are normally located in the stroma of most organs and during the formation of a tumor (the development of inflammation and fibrosis in tumors), and they “activate” (begin to produce various components of the extracellular matrix, matrix metalloproteinases, leading to the degradation of the extracellular matrix, etc.) and at the same time transform into tumor-associated fibroblasts, which are the basis of the tumor stroma (provide regulatory, nutritional, and “skeleton” functions for the tumor), which continuously interact with tumor cells, contributing to each other’s development, and ultimately leading to tumor progression [80,81]. Tumor-associated fibroblasts, causing the development of a “desmoplastic reaction” (a large number of collagens of types I, III, IV, V, fibrinolytic protein, laminin, and hyaluronic acid are formed, and various proteases and matrix metalloproteinases are secreted, which lead to a pronounced remodeling of the extracellular matrix, etc.), determine the formation of the skeleton structure of the tumor (tissue hardening occurs and fibrosis of stromal cells), which underlies the evasion of the tumor from immunity (prevents the penetration of immune cells) and provides an “optimal” environment for interaction between tumor cells and cytokines, increasing migration and the invasion of cancer cells, thereby contributing to the progression of the tumor, including in BC [81,82]. So, the significant functional effects of SHBG-significant BC-associated loci in fibroblasts, in relation to the 18 genes that we have established, can be the biomedical basis for the involvement of fibroblasts in the pathogenesis of BC, due to the regulatory effects of the loci controlling the formation of SHBG in the organism. Interestingly, an earlier study of BC performed on the same sample of patients/controls showed significant associations of a number of functionally significant polymorphic loci of the matrix metalloproteinase 9 gene (rs17576 and rs2250889) in the formation of the disease [11].

For some limitations of this study, the following points can be highlighted: (a) the functional effects of BC-related loci assumed in the work based on in silico analysis need in vivo/in vitro experimental confirmation; (b) in this work, the levels of SHBG and sex hormones (testosterone, estrogens, etc.) were not determined, which would allow it to be more convincing to show that the biological pathways underlying the associations of the SNP candidate gene SHBG with BC.

In the framework of further prospects for the development of research on this topic (in addition to the above limitations of this study and which need further study), the following should be noted: (a) it is necessary to analyze in more detail (using more numerous samples of patients and controls) the a priori presumed features of the association of SNP candidate gene SHBG with BC of different biological subtypes, BMI-dependent associations, etc.; (b) to consider the joint contribution of genetic determinants that determine both the level of SHBG and the level of BC-significant sex hormones (estrogens, testosterone, etc.) in the organism; (c) based on the recently obtained new GWAS data on the genetic determinants of SHBG [72], in the future it is necessary to conduct more extensive genetic and epidemiological studies of BC with the inclusion of all (or most of them) currently known SNP candidate genes of SHBG.

## 4. Materials and Methods

### 4.1. Study Subjects

The present genetic study was supported by the “Ethics Committee (Human Investigation)” of the Belgorod State National Research University, and was based on the mandatory receipt of informed consent (certified by a personal signature) from each participant of the study. The sample was formed during 2010–2016 on the basis of two specialized hospitals of the Belgorod region, such as an Oncological Dispensary (BC) and a Regional Clinical Hospital (non BC). As a result of 7 years of collecting the material, a sample of 358 BC patients and 1140 control (non BC) of Russian women were born and lived in Central Russia was formed [83]. The group of patients included women with a confirmed diagnosis of BC by a generally accepted morphological method (the study was performed by certified morphologists-pathologists [84]). The control group women were cancer-free (they did not have any tumors at the time of examination and in the anamnesis) and did not suffer from any serious diseases [85]).

All the considered phenotypic characteristics of the subjects (BC/non BC) are shown in Table 7 (these data were presented in our previously published work [11]). Statistically significant higher BMI (*p* = 0.0001) and a higher proportion of obese individuals (*p* = 0.0006) were found when comparing “BC vs. Controls”, which was the basis for using these characteristics (together with the age of women) as confounders when evaluating associations between SNP and BC [11]. It is essential that the studied subjects (68.16%[BC], 63.60%[Controls], *p* = 0.13) were dominated by woman in postmenopausal status.

### 4.2. DNA Analysis (SNP Selection/Genotyping)

All DNA samples used in the experimental study were previously isolated from venous blood by the standard method (the widespread method of “phenol-chloroform-alcohol” DNA extraction was used [86]). For PCR detection of polymorphisms (carried out on a CFX96 device that allows evaluating the results in real time [87]), DNA samples with parameters such as concentration—10–20 ng/mL and purity (absorption at wavelengths of 260 nm and 280 nm, 260/280 nm)—1.7–2.0 were used (DNA samples were tested on microvolume spectrophotometer NanoDrop™ 2000 (Thermo Fisher Scientific Inc., Waltham, MA, USA) [88].

To solve the problem set in the work, we carried out genotyping of 9 SNPs associated with the content of SHBG in the human organism (previously obtained GWAS materials, Supplementary Table S15) [23,72,74,89,90,91]. To search for associations with BC, such loci as rs780093 *GCKR*, rs17496332 *PRMT6*, rs3779195 *BAIAP2L1*, rs10454142 *PPP1R21*, rs7910927 *JMJD1C*, rs4149056 *SLCO1B1*, rs440837 *ZBTB10*, rs12150660 *SHBG*, rs8023580 *NR2F2* were studied. The quality of the experimental data obtained (the probability of incorrect genotyping) was checked with additional repeated genotyping of 3–5% of DNA samples (the format of “blind” re-genotyping was used [92]; the results obtained as a result of this procedure (genotyping errors were no more than 1%) allow us to consider the data obtained during laboratory studies acceptable for statistical (genetic) analysis.

### 4.3. Genetic Data Statistical Analysis

Before analyzing the SNP-BC associations, we performed a check for the presence/absence of differences between the registered/expected (in accordance with the parameters of the Hardy–Weinberg rule) dispensation of genotypes at all loci in BC and non-BC cohorts. The SNP-BC correlations (with calculation of such parameters association link as the OR [odd ratio] and 95%CI [confidence intervals] for OR [93])) were revealed by the logistic regression (4 models of allelic gene variants [minor/major alleles SNP] interaction were considered such as dominant/recessive/additive/allelic [94]) in the PLINK program (Java-linked version 1.07) [95] when correcting for confounders (age; BMI; proportion of obese) and multiple comparisons (permutation method [the p_perm_ indicator was calculated based on adaptive test] was applied [96]) and the calculation of the power of significant associations (Quanto tool was used [97]).

The multiSNP-BC correlations (for discovery BC-risky of non-allelic gene variants interactions) were detected by the MB-MDR logistic regression [98]. The MB-MDR analysis used the necessary confounders (age; BMI; proportion of obese) and a given number of permutations equal to 1000. To accomplish the permutation procedure, we handpicked the very significant non-allelic gene interaction models for BC risk that met the threshold value *p* that we introduced (for its calculation, we used data on the number of all possible combinations of 9 considered genetic markers at different levels of their combination, i.e., we used the Bonferroni correction). We used the following “threshold” levels of *p_MB-MDR_*: *p* = 0.05/36 = 1.39 × 10^−3^ (2 SNP interaction); *p* = 0.05/84 = 5.95 × 10^−4^ (3 SNP interaction); *p* = 0.05/126 = 3.97 × 10^−4^ (4 SNP interaction); *p* = 0.05/126 = 3.97 × 10^−4^ (5 SNP interaction) [99]. A *p*_perm_ value of at least/equal 0.05 (for allelic gene variants interactions [100]) and 0.001 (for non-allelic gene variants interactions [101]) were the reason to consider the differences reliable. Visualization of phenotypic BC-significant effects (% of BC entropy; communication orientation) of allelic/non-allelic gene variant interactions was fulfilled in the MDR program [102,103,104].

### 4.4. In Silico Testing of Possible Functionality of BC-Involved SNPs/Genes

The final stage of the work was devoted in silico to predicting the possible functionality of BC-involved SNPs and proxy variants (r^2^ ≥ 0.80) [105] using a fairly large range of databases widely used in biological research [106,107,108,109], such as GTExportal (accessed on 21 September 2023) [110]; QTLbase (accessed on 21 September 2023) [111]; HaploReg (accessed on 13 September 2023) [112]; regBase-CAN (accessed on 22 September 2023) [113]; SIFT (accessed on 21 September 2023) [114]; STRING (accessed on 14 October 2023) [115]; Poly-Phen2 (accessed on 21 September 2023) [116]; Gene Ontology (accessed on 14 October 2023) [117].

## 5. Conclusions

GWAS-impacted SHBG-correlated gene polymorphisms are associated with BC risk in Caucasian women of Russia.

## Figures and Tables

**Table 1:** Associations of the SHBG-impacted gene polymorphisms with breast cancer.

SNP	Gene	Minor Allele	n	Allelic Model				Additive Model				Dominant Model				Recessive Model			
				OR	95%CI		P	OR	95%CI		P	OR	95%CI		P	OR	95%CI		P
					L95	U95			L95	U95			L95	U95			L95	U95	
rs17496332	*PRMT6*	G	1422	0.94	0.79	1.12	0.502	0.99	0.80	1.23	0.958	0.89	0.66	1.02	0.438	1.24	0.81	1.88	0.320
rs780093	*GCKR*	T	1445	0.96	0.80	1.14	0.605	0.90	0.72	1.12	0.350	0.92	0.68	1.26	0.622	0.79	0.52	1.02	0.267
rs10454142	*PPP1R21*	C	1424	1.16	0.97	1.39	0.099	**1.31**	**1.08**	**1.65**	**0.022**	1.32	0.97	1.80	0.072	1.51	0.93	2.48	0.098
rs3779195	*BAIAP2L1*	A	1421	1.06	0.85	1.32	0.626	1.07	0.82	1.41	0.609	1.15	0.84	1.59	0.384	0.73	0.30	1.76	0.486
rs440837	*ZBTB10*	G	1408	0.92	0.75	1.13	0.426	0.93	0.72	1.19	0.543	0.81	0.59	1.09	0.166	1.46	0.82	2.60	0.203
rs7910927	*JMJD1C*	T	1446	0.93	0.78	1.10	0.381	0.94	0.76	1.16	0.571	0.88	0.63	1.24	0.469	0.97	0.68	1.38	0.852
rs4149056	*SLCO1B1*	C	1385	0.91	0.74	1.12	0.373	1.00	0.76	1.30	0.977	0.99	0.73	1.36	0.962	1.01	0.48	2.13	0.975
rs8023580	*NR2F2*	C	1440	0.89	0.74	1.08	0.253	0.91	0.72	1.16	0.444	0.97	0.72	1.31	0.838	0.63	0.34	1.18	0.149
rs12150660	*SHBG*	T	1452	1.00	0.82	1.21	0.983	0.94	0.74	1.20	0.635	0.93	0.69	1.25	0.624	0.94	0.52	1.69	0.832

Note: All results were obtained after adjustment for covariates; OR odds ratio; 95%CI, 95% confidence interval; Statistically significant values and *p* values < 0.05 are shown in bold.

**Table 2:** SNP × SNP interactions of SHBG-impacted genes significantly associated with breast cancer.

N	SNP × SNP Interaction Models	NH	*beta*H	WH	NL	*beta*L	WL	p_perm_
Two-order interaction models (*p* < 1.23 × 10^−4^)								
1	rs4149056 *SLCO1B1*-rs10454142 *PPP1R21*	3	0.696	16.39	0	-	-	<0.001
2	rs440837 *ZBTB10*-rs10454142 *PPP1R21*	3	0.572	14.74	1	−0.334	4.23	0.001
Three-order interaction models (*p* < 1.64 × 10^−6^)								
1	rs7910927 *JMJD1C*—rs440837 *ZBTB10*-rs10454142 *PPP1R21*	2	1.734	25.32	1	−0.642	3.35	<0.001
2	rs4149056 *SLCO1B1*—rs440837 *ZBTB10*-rs10454142 *PPP1R21*	3	0.967	22.98	1	−0.316	2.77	<0.001
Four-order interaction models (*p* < 4.65 × 10^−9^)								
1	rs4149056 *SLCO1B1*-rs440837 *ZBTB10*-rs10454142 *PPP1R21*-rs780093 *GCKR*	6	1.537	41.44	2	−0.574	5.60	<0.001
2	rs8023580 *NR2F2*-rs7910927 *JMJD1C*-rs10454142 *PPP1R21*-rs17496332 *PRMT6*	7	1.345	34.33	2	−0.749	6.68	<0.001
3	rs7910927 *JMJD1C*-rs10454142 *PPP1R21*-rs780093 *GCKR*-rs17496332 *PRMT6*	7	1.575	37.59	2	−0.878	11.36	<0.001
Five-order interaction models (*p* = 4.79 × 10^−12^)								
1	rs7910927 *JMJD1C*-rs3779195 *BAIAP2L1*-rs10454142 *PPP1R21*-rs780093 *GCKR*-rs17496332 *PRMT6*	10	1.791	51.58	2	−0.997	10.57	<0.001
2	rs8023580 *NR2F2*-rs7910927 *JMJD1C*-rs10454142 *PPP1R21*-rs780093 *GCKR*-rs17496332 *PRMT6*	11	1.876	47.77	2	−2.118	6.95	<0.001

Note: The results were obtained using the MB-MDR method, with adjustment for covariates. NH, number of significant high risk genotypes in the interaction; beta H, regression coefficient for high risk exposition in the step2 analysis; WH, Wald statistic for high risk category; NL, number of significant low risk genotypes in the interaction; beta L, regression coefficient for low risk exposition in the step2 analysis; WL, Wald statistic for low risk category; p_perm_, permutation *p*-value for the interaction model (1000 permutations).

**Table 3:** Estimated epigenetic potential of the BC-associated locus rs10454142 *PPP1R21* and proxy SNP (r^2^ ≥ 0.80) (in silico data of Haploreg).

SNP (Position hg38) (r^2^, LD)	Liver	Mammary Gland		Transcription Factors
		Breast Variant Human Mammary Epithelial Cells	Breast Myoepithelial Primary Cells	
rs78597273 (48380665) (r^2^ = 0.81, LD = 0.99)		H3K4me1_Enh	H3K4me1_Enh	MIZF
rs11689645 (48381420) (r^2^ = 0.81, LD = 0.99)		H3K4me1_Enh		AP-1, AP-2, BAF155, BATF, Myc, GR, BCL, Bach1, Bach2, ATA, HMGN3, KAP1, Maf, NF-E2, STAT, PRDM1, TCF4, p300
rs111960813 (48404376) (r^2^ = 0.80, LD = 0.93)				ELF1, Myc, ZBRK1
rs56391806 (48404838) (r^2^ = 0.85, LD = 0.98)				Fox, Hoxb6
rs55744465 (48405316) (r^2^ = 0.85, LD = 0.98)				Hoxa5
rs201414717 (48419259) (r^2^ = 1.00, LD = 1.00)	H3K4me1_Enh H3K4me3_Pro H3K27ac_Enh H3K9ac_Pro	H3K4me1_Enh		AP-4, CACD, WT1, YY1, TAL1, TCF12, Rad21, LBP-1, ZNF219
**rs10454142** **(48419260)**	**H3K4me1_Enh** **H3K4me3_Pro** **H3K27ac_Enh** **H3K9ac_Pro**	**H3K4me1_Enh**		**NF-kappaB**
rs10454143 (48419261) (r^2^ = 1.00, LD = 1.00)	H3K4me1_Enh H3K4me3_Pro H3K27ac_Enh H3K9ac_Pro			Barx1, CEBPD, Hoxa3
rs4638844 (48427445) (r^2^ = 0.81, LD = 0.94)			H3K9ac_Pro	CIZ, FAC1, Foxa, Foxd3, Foxj2, Foxk1, Foxo, Foxp1, HDAC2, Irf, Pax-4, Sox, p300, RREB-1, Zfp105

Note: H3K4me1_Enh, SNP location in the region of H3K4me1 histones-marking enhancers; H3K27ac_Enh, active enhancers; H3K4me3_Pro, promoters; H3K9ac_Pro, active promoters; bold highlights, BC-causal SNP.

**Table 4:** Associations of rs10454142 *PPP1R21* and strongly coupled SNPs (r^2^ ≥ 0.80) with expression (eQTL) and alternative splicing (sQTL) of genes in the organism (in total), liver, mammary gland, and fibroblasts (in silico data of GTE x portal).

SNP (Position, hg38) (r^2^, LD)	eQTL				sQTL		
	In the Organism (In Total)	Liver	Mammary Gland	Fibroblasts	In The Organism (In Total)	Mammary Gland	Fibroblasts
rs17855177 (48375113) (r^2^ = 0.81, LD = 0.99)	*FOXN2*, *FSHR*, *GTF2A1L*, *LHCGR*, *MSH6*, *PPP1R21*, *RP11-191L17.1*, *RP11-460M2.1*, *STON1*, *STON1-GTF2A1L*	*GTF2A1L*	*GTF2A1L*, *PPP1R21*, *STON1-GTF2A1L*, *RP11-460M2.1*	*GTF2A1L*, *PPP1R21*, *MSH6*	*GTF2A1L*, *PPP1R21*, *STON1*, *STON1-GTF2A1L*	*GTF2A1L*, *PPP1R21*, *STON1*	*PPP1R21*
rs78597273 (48380665) (r^2^ = 0.81, LD = 0.99)	*FOXN2*, *FSHR*, *GTF2A1L*, *LHCGR*, *MSH6*, *PPP1R21*, *RP11-191L17.1*, *RP11-460M2.1*, *STON1*, *STON1-GTF2A1L*	*GTF2A1L*	*GTF2A1L*, *PPP1R21*, *STON1-GTF2A1L*, *RP11-460M2.1*	*GTF2A1L*, *PPP1R21*, *MSH6*	*GTF2A1L*, *PPP1R21*, *STON1*, *STON1-GTF2A1L*	*GTF2A1L*, *PPP1R21*, *STON1*	*PPP1R21*
rs11689645 (48381420) (r^2^ = 0.81, LD = 0.99)	*FOXN2*, *FSHR*, *GTF2A1L*, *LHCGR*, *MSH6*, *PPP1R21*, *RP11-191L17.1*, *RP11-460M2.1*, *STON1*, *STON1-GTF2A1L*	*GTF2A1L*	*GTF2A1L*, *PPP1R21*, *STON1-GTF2A1L*, *RP11-460M2.1*	*GTF2A1L*, *PPP1R21*, *MSH6*	*GTF2A1L*, *PPP1R21*, *STON1*, *STON1-GTF2A1L*	*GTF2A1L*, *PPP1R21*, *STON1*	*PPP1R21*
rs111960813 (48404376) (r^2^ = 0.80, LD = 0.93)	*FOXN2*, *FSHR*, *GTF2A1L*, *LHCGR*, *MSH6*, *PPP1R21*, *RP11-191L17.1*, *RP11-460M2.1*, *STON1*, *STON1-GTF2A1L*	*GTF2A1L*	*GTF2A1L*, *PPP1R21*, *STON1-GTF2A1L*, *RP11-460M2.1*, *MSH6*	*GTF2A1L*, *PPP1R21*, *MSH6*, *FOXN2*	*GTF2A1L*, *PPP1R21*, *STON1*, *STON1-GTF2A1L*	*GTF2A1L*, *PPP1R21*, *STON1*	*PPP1R21*
rs56391806 (48404838) (r^2^ = 0.85, LD = 0.98)	*FOXN2*, *FSHR*, *GTF2A1L*, *LHCGR*, *MSH6*, *PPP1R21*, *RP11-460M2.1*, *STON1-GTF2A1L*	*GTF2A1L*	*GTF2A1L*, *PPP1R21*, *STON1-GTF2A1L*, *RP11-460M2.1*, *MSH6*	*GTF2A1L*, *PPP1R21*, *MSH6*, *FOXN2*	*GTF2A1L*, *PPP1R21*, *STON1*, *STON1-GTF2A1L*	*GTF2A1L*, *PPP1R21*, *STON1*	*PPP1R21*
rs55744465 (48405316) (r^2^ = 0.85, LD = 0.98)	*FOXN2*, *FSHR*, *GTF2A1L*, *LHCGR*, *MSH6*, *PPP1R21*, *RP11-460M2.1*, *STON1*, *STON1-GTF2A1L*	*GTF2A1L*	*GTF2A1L*, *PPP1R21*, *STON1-GTF2A1L*, *RP11-460M2.1*, *MSH6*	*GTF2A1L*, *PPP1R21*, *MSH6*, *FOXN2*	*GTF2A1L*, *PPP1R21*, *STON1*, *STON1-GTF2A1L*	*GTF2A1L*, *PPP1R21*, *STON1*	*PPP1R21*
**rs10454142 (48419260) (r^2^ = 1.00, LD = 1.00)**	***FOXN2*, *FSHR*, *GTF2A1L*, *LHCGR*, *MSH6*, *PPP1R21*, *RP11-460M2.1*, *STON1*, *STON1-GTF2A1L***	***GTF2A1L***	***GTF2A1L*, *PPP1R21*, *STON1-GTF2A1L*, *RP11-460M2.1*, *MSH6***	***GTF2A1L*, *PPP1R21*, *MSH6*, *FOXN2***	***GTF2A1L*, *PPP1R21*, *STON1*, *STON1-GTF2A1L***	***GTF2A1L*, *PPP1R21*, *STON1***	***PPP1R21***
rs10454143 (48419261) (r^2^ = 1.00, LD = 1.00)	*FOXN2*, *FSHR*, *GTF2A1L*, *LHCGR*, *MSH6*, *PPP1R21*, *RP11-460M2.1*, *STON1*, *STON1-GTF2A1L*	*GTF2A1L*	*GTF2A1L*, *PPP1R21*, *STON1-GTF2A1L*, *RP11-460M2.1*, *MSH6*	*GTF2A1L*, *PPP1R21*, *MSH6*, *FOXN2*	*GTF2A1L*, *PPP1R21*, *STON1*, *STON1-GTF2A1L*	*GTF2A1L*, *PPP1R21*, *STON1*	*PPP1R21*
rs13399936 (48426987) (r^2^ = 0.87, LD = 0.96)	*FOXN2*, *FSHR*, *GTF2A1L*, *LHCGR*, *MSH6*, *PPP1R21*, *RP11-191L17.1*, *RP11-460M2.1*, *STON1*, *STON1-GTF2A1L*	*GTF2A1L*	*GTF2A1L*, *PPP1R21*, *STON1-GTF2A1L*, *RP11-460M2.1*	*GTF2A1L*, *PPP1R21*	*GTF2A1L*, *PPP1R21*, *STON1*, *STON1-GTF2A1L*	*GTF2A1L*, *PPP1R21*, *STON1*	*PPP1R21*

Note: Bold highlights, BC-causal SNP.

**Table 5:** Localization of BC-associated SHBG candidate genes and SNPs in regions of histone proteins marking enhancers (H3K4me1), promoters (H3K4me3), active enhancers (H3K27ac), and active promoters (H3K9ac) in breast and liver cell lines (data HaploReg).

SNP	Breast Variant Human Mammary Epithelial Cells (vHMEC)	Breast Myoepithelial Primary Cells	Liver
rs17496332 *PRMT6*		H3K9ac_Pro	
rs780093 *GCKR*		H3K9ac_Pro	H3K4me1_Enh H3K4me3_Pro H3K27ac_Enh
rs10454142 *PPP1R21*	H3K4me1_Enh		H3K4me1_Enh H3K4me3_Pro H3K27ac_Enh H3K9ac_Pro
rs440837 *ZBTB10*			H3K4me1_Enh H3K4me3_Pro H3K27ac_Enh H3K9ac_Pro
rs7910927 *JMJD1C*			H3K4me1_Enh
rs8023580 *NR2F2*			H3K4me1_Enh

Note: H3K4me1_Enh, SNP location in the region of H3K4me1 histones marking enhancers; H3K27ac_Enh, active enhancers; H3K4me3_Pro, promoters; H3K9ac_Pro, active promoters.

**Table 6:** Prognostic value of BC-associated candidate genes SHBG SNP as drivers of tumor development (regBase-CAN data).

SNP	Score	Phred Score	Potential Role
rs17496332 *PRMT6*	0.0319	1.3145	
rs780093 *GCKR*	0.3656	4.2654	
**rs10454142** ***PPP1R21***	**0.7992**	**7.0231**	**likely cancer driver**
rs3779195 *BAIAP2L1*	0.0107	0.6073	
rs440837 *ZBTB10*	0.0190	0.9416	
rs7910927 *JMJD1C*	0.0560	1.8015	
**rs4149056 *SLCO1B1***	**0.9834**	**12.6570**	**likely cancer driver**
rs8023580 *NR2F2*	0.1781	3.1007	

Note: Significant values are shown in bold.

**Table 7:** Phenotypic characteristics of the study participants.

Parameters	BC pPatients, % (n)	Controls, % (n)	*p*
*N*	358	1140	-
Age, years (min–max)	55.74 ± 12.79 (28–84)	55.02 ± 12.35 (29–80)	0.17
<50 years	33.80 (121)	37.81 (431)	0.19
≥50 years	66.20 (237)	62.19 (709)	
BMI, kg/m^2^	30.27 ± 6.13	27.64 ± 5.42	0.0001
Obesity (BMI ≥ 30) (yes)	33.24 (119)	22.19 (253)	0.0006
Age at menarche, years	12.42 ± 1.12	12.58 ± 1.13	0.51
Age at menopause, years	48.27 ± 5.02	47.88 ± 4.82	0.34
Mensuration status			
Premenopause	31.84 (114)	36.40 (415)	0.13
Postmenopause	68.16 (244)	63.60 (725)	
Smoker (yes)	22.07 (79)	19.04 (217)	0.22
Clinicopathological parameters of BC patients			
Stage of cancer	T_0_–T_2_—74%, T_3_–T_4_—26%		
Lymph node involvement (N)	negative—47%, positive—53%		
Estrogen receptor (ER)	negative—34%, positive—66%		
Progesterone receptor (PR)	negative—41%, positive—59%		
Human epidermal growth factor receptor 2 (HER2)	negative—64%, positive—36%		
Tumor histological type	ductal—94%, lobular—6%		
Tumor histological grade (G)	G1/G2—68%, G3—32%		
Progression	absent—66%, present—34%		
Metastasis	absent—78%, present—22%		
Death	absent—81%, present—19%		

Note: G1, well differentiated; G2, moderately differentiated; G3, poorly differentiated.

## Data Availability

The data generated in the present study are available from the corresponding author upon reasonable request.

## Supplementary Materials

The following supporting information can be downloaded at: https://www.mdpi.com/article/10.3390/ijms25042182/s1.

## References

[1] Ferlay J., Colombet M., Soerjomataram I., Parkin D.M., Piñeros M., Znaor A., Bray F. (2021). Cancer statistics for the year 2020: An overview. Int. J. Cancer.

[2] Sung H., Ferlay J., Siegel R.L., Laversanne M., Soerjomataram I., Jemal A., Bray F. (2021). Global cancer statistics 2020: GLOBOCAN estimates of incidence and mortality worldwide for 36 cancers in 185 countries. CA Cancer J. Clin..

[3] Arnold M., Morgan E., Rumgay H., Mafra A., Singh D., Laversanne M., Vignat J., Gralow J.R., Cardoso F., Siesling S. (2022). Current and future burden of breast cancer: Global statistics for 2020 and 2040. Breast.

[4] Shiovitz S., Korde L.A. (2015). Genetics of breast cancer: A topic in evolution. Ann. Oncol..

[5] Möller S., Mucci L.A., Harris J.R., Scheike T., Holst K., Halekoh U., Adami H.O., Czene K., Christensen K., Holm N.V. (2016). The Heritability of Breast Cancer among Women in the Nordic Twin Study of Cancer. Cancer Epidemiol. Biomark. Prev..

[6] Mucci L.A., Hjelmborg J.B., Harris J.R., Czene K., Havelick D.J., Scheike T., Graff R.E., Holst K., Möller S., Unger R.H. (2016). Nordic Twin Study of Cancer (NorTwinCan) Collaboration. Familial Risk and Heritability of Cancer among Twins in Nordic Countries. JAMA.

[7] Michailidou K., Lindström S., Dennis J., Beesley J., Hui S., Kar S., Lemaçon A., Soucy P., Glubb D., Rostamianfar A. (2017). Association analysis identifies 65 new breast cancer risk loci. Nature.

[8] Lilyquist J., Ruddy K.J., Vachon C.M., Couch F.J. (2018). Common Genetic Variation and Breast Cancer Risk-Past, Present, and Future. Cancer Epidemiol. Biomark. Prev..

[9] Shu X., Long J., Cai Q., Kweon S.S., Choi J.Y., Kubo M., Park S.K., Bolla M.K., Dennis J., Wang Q. (2020). Identification of novel breast cancer susceptibility loci in meta-analyses conducted among Asian and European descendants. Nat. Commun..

[10] Adedokun B., Du Z., Gao G., Ahearn T.U., Lunetta K.L., Zirpoli G., Figueroa J., John E.M., Bernstein L., Zheng W. (2021). Cross-ancestry GWAS meta-analysis identifies six breast cancer loci in African and European ancestry women. Nat. Commun..

[11] Pavlova N., Demin S., Churnosov M., Reshetnikov E., Aristova I., Churnosova M., Ponomarenko I. (2022). Matrix Metalloproteinase Gene Polymorphisms Are Associated with Breast Cancer in the Caucasian Women of Russia. Int. J. Mol. Sci..

[12] Trabert B., Sherman M.E., Kannan N., Stanczyk F.Z. (2020). Progesterone and Breast Cancer. Endocr. Rev..

[13] Tin Tin S., Reeves G.K., Key T.J. (2021). Endogenous hormones and risk of invasive breast cancer in pre- and post-menopausal women: Findings from the UK Biobank. Br. J. Cancer.

[14] Chen F., Wen W., Long J., Shu X., Yang Y., Shu X.O., Zheng W. (2022). Mendelian randomization analyses of 23 known and suspected risk factors and biomarkers for breast cancer overall and by molecular subtypes. Int. J. Cancer.

[15] Tang S.N., Zuber V., Tsilidis K.K. (2022). Identifying and ranking causal biochemical biomarkers for breast cancer: A Mendelian randomisation study. BMC Med..

[16] Key T.J., Appleby P.N., Reeves G.K., Travis R.C., Brinton L.A., Helzlsouer K.J., Dorgan J.F., Gapstur S.M., Gaudet M.M., Kaaks R. (2015). Steroid hormone measurements from different types of assays in relation to body mass index and breast cancer risk in postmenopausal women: Reanalysis of eighteen prospective studies. Steroids.

[17] Coviello A.D., Zhuang W.V., Lunetta K.L., Bhasin S., Ulloor J., Zhang A., Karasik D., Kiel D.P., Vasan R.S., Murabito J.M. (2011). Circulating testosterone and SHBG concentrations are heritable in women: The Framingham Heart Study. J. Clin. Endocrinol. Metab..

[18] Hammond G.L. (2016). Plasma steroid-binding proteins: Primary gatekeepers of steroid hormone action. J. Endocrinol..

[19] Balogh A., Karpati E., Schneider A.E., Hetey S., Szilagyi A., Juhasz K., Laszlo G., Hupuczi P., Zavodszky P., Papp Z. (2019). Sex hormone-binding globulin provides a novel entry pathway for estradiol and influences subsequent signaling in lymphocytes via membrane receptor. Sci. Rep..

[20] Sinnott-Armstrong N., Naqvi S., Rivas M., Pritchard J.K. (2021). GWAS of three molecular traits highlights core genes and pathways alongside a highly polygenic background. eLife.

[21] He X.Y., Liao Y.D., Yu S., Zhang Y., Wang R. (2015). Sex hormone binding globulin and risk of breast cancer in postmenopausal women: A meta-analysis of prospective studies. Horm. Metab. Res..

[22] Varghese J.S., Smith P.L., Folkerd E., Brown J., Leyland J., Audley T., Warren R.M., Dowsett M., Easton D.F., Thompson D.J. (2012). The heritability of mammographic breast density and circulating sex-hormone levels: Two independent breast cancer risk factors. Cancer Epidemiol. Biomark. Prev..

[23] Coviello A.D., Haring R., Wellons M., Vaidya D., Lehtimäki T., Keildson S., Lunetta K.L., He C., Fornage M., Lagou V. (2012). A genome-wide association meta-analysis of circulating sex hormone-binding globulin reveals multiple Loci implicated in sex steroid hormone regulation. PLoS Genet..

[24] Dimou N.L., Papadimitriou N., Gill D., Christakoudi S., Murphy N., Gunter M.J., Travis R.C., Key T.J., Fortner R.T., Haycock P.C. (2019). Sex hormone binding globulin and risk of breast cancer: A Mendelian randomization study. Int. J. Epidemiol..

[25] Becchis M., Frairia R., Ferrera P., Fazzari A., Ondei S., Alfarano A., Coluccia C., Biglia N., Sismondi P., Fortunati N. (1999). The additionally glycosylated variant of human sex hormone-binding globulin (SHBG) is linked to estrogen-dependence of breast cancer. Breast Cancer Res. Treat..

[26] Dunning A.M., Dowsett M., Healey C.S., Tee L., Luben R.N., Folkerd E., Novik K.L., Kelemen L., Ogata S., Pharoah P.D. (2004). Polymorphisms associated with circulating sex hormone levels in postmenopausal women. J. Natl. Cancer Inst..

[27] Cui Y., Shu X.O., Cai Q., Jin F., Cheng J.R., Cai H., Gao Y.T., Zheng W. (2005). Association of breast cancer risk with a common functional polymorphism (Asp327Asn) in the sex hormone-binding globulin gene. Cancer Epidemiol. Biomark. Prev..

[28] Thompson D.J., Healey C.S., Baynes C., Kalmyrzaev B., Ahmed S., Dowsett M., Folkerd E., Luben R.N., Cox D., Ballinger D. (2008). Identification of common variants in the SHBG gene affecting sex hormone-binding globulin levels and breast cancer risk in postmenopausal women. Cancer Epidemiol. Biomark. Prev..

[29] Iwasaki M., Hamada G.S., Nishimoto I.N., Netto M.M., Motola J., Laginha F.M., Kasuga Y., Yokoyama S., Onuma H., Nishimura H. (2010). Dietary isoflavone intake, polymorphisms in the CYP17, CYP19, 17beta-HSD1, and SHBG genes, and risk of breast cancer in case-control studies in Japanese, Japanese Brazilians, and non-Japanese Brazilians. Nutr. Cancer.

[30] Zhang B., Beeghly-Fadiel A., Lu W., Cai Q., Xiang Y.B., Zheng Y., Long J., Ye C., Gu K., Shu X.O. (2011). Evaluation of functional genetic variants for breast cancer risk: Results from the Shanghai breast cancer study. Am. J. Epidemiol..

[31] Zhou J.Y., Shi R., Yu H.L., Zheng W.L., Ma W.L. (2012). Association between SHBG Asp327Asn (rs6259) polymorphism and breast cancer risk: A meta-analysis of 10,454 cases and 13,111 controls. Mol. Biol. Rep..

[32] Nyante S.J., Gammon M.D., Kaufman J.S., Bensen J.T., Lin D.Y., Barnholtz-Sloan J.S., Hu Y., He Q., Luo J., Millikan R.C. (2015). Genetic variation in estrogen and progesterone pathway genes and breast cancer risk: An exploration of tumor subtype-specific effects. Cancer Causes Control.

[33] Pan Z., Fu Z., Song Q., Cao W., Cheng W., Xu X. (2016). Genetic polymorphisms and haplotype of hormone-related genes are associated with the risk of breast cancer in Chinese women. Genet. Mol. Res..

[34] Sakaue S., Kanai M., Tanigawa Y., Karjalainen J., Kurki M., Koshiba S., Narita A., Konuma T., Yamamoto K., Akiyama M. (2021). A cross-population atlas of genetic associations for 220 human phenotypes. Nat. Genet..

[35] Chen V.L., Du X., Chen Y., Kuppa A., Handelman S.K., Vohnoutka R.B., Peyser P.A., Palmer N.D., Bielak L.F., Halligan B. (2021). Genome-wide association study of serum liver enzymes implicates diverse metabolic and liver pathology. Nat. Commun..

[36] Pazoki R., Vujkovic M., Elliott J., Evangelou E., Gill D., Ghanbari M., van der Most P.J., Pinto R.C., Wielscher M., Farlik M. (2021). Genetic analysis in European ancestry individuals identifies 517 loci associated with liver enzymes. Nat. Commun..

[37] Plotnikov D.Y. (2023). Mendelian randomization: Using genetic information in epidemiological studies (review). Res. Results Biomed..

[38] Hammond G.L. (2011). Diverse roles for sex hormone-binding globulin in reproduction. Biol. Reprod..

[39] Dunn J.F., Nisula B.C., Rodbard D. (1981). Transport of steroid hormones: Binding of 21 endogenous steroids to both testosterone-binding globulin and corticosteroid-binding globulin in human plasma. J. Clin. Endocrinol. Metab..

[40] Qu X., Donnelly R. (2020). Sex Hormone-Binding Globulin (SHBG) as an Early Biomarker and Therapeutic Target in Polycystic Ovary Syndrome. Int. J. Mol. Sci..

[41] Key T., Appleby P., Barnes I., Reeves G., Endogenous Hormones and Breast Cancer Collaborative Group (2002). Endogenous sex hormones and breast cancer in postmenopausal women: Reanalysis of nine prospective studies. J. Natl. Cancer Inst..

[42] Fortunati N., Catalano M.G. (2006). Sex hormone-binding globulin (SHBG) and estradiol cross-talk in breast cancer cells. Horm. Metab. Res..

[43] Fortunati N., Catalano M.G., Boccuzzi G., Frairia R. (2010). Sex Hormone-Binding Globulin (SHBG), estradiol and breast cancer. Mol. Cell. Endocrinol..

[44] Arthur R.S., Xue X., Rohan T.E. (2020). Prediagnostic Circulating Levels of Sex Steroid Hormones and SHBG in Relation to Risk of Ductal Carcinoma In Situ of the Breast among UK Women. Cancer Epidemiol. Biomark. Prev..

[45] Drummond A.E., Swain C.T.V., Brown K.A., Dixon-Suen S.C., Boing L., van Roekel E.H., Moore M.M., Gaunt T.R., Milne R.L., English D.R. (2022). Linking Physical Activity to Breast Cancer via Sex Steroid Hormones, Part 2: The Effect of Sex Steroid Hormones on Breast Cancer Risk. Cancer Epidemiol. Biomark. Prev..

[46] Secreto G., Girombelli A., Krogh V. (2019). Androgen excess in breast cancer development: Implications for prevention and treatment. Endocr. Relat. Cancer.

[47] Vasiliou S.K., Diamandis E.P. (2019). Androgen receptor: A promising therapeutic target in breast cancer. Crit. Rev. Clin. Lab. Sci..

[48] Venema C.M., Bense R.D., Steenbruggen T.G., Nienhuis H.H., Qiu S.Q., van Kruchten M., Brown M., Tamimi R.M., Hospers G.A.P., Schröder C.P. (2019). Consideration of breast cancer subtype in targeting the androgen receptor. Pharmacol. Ther..

[49] Chen M., Yang Y., Xu K., Li L., Huang J., Qiu F. (2020). Androgen Receptor in Breast Cancer: From Bench to Bedside. Front. Endocrinol..

[50] Gerratana L., Basile D., Buono G., De Placido S., Giuliano M., Minichillo S., Coinu A., Martorana F., De Santo I., Del Mastro L. (2018). Androgen receptor in triple negative breast cancer: A potential target for the targetless subtype. Cancer Treat. Rev..

[51] Felty Q., Xiong W.C., Sun D., Sarkar S., Singh K.P., Parkash J., Roy D. (2005). Estrogen-induced mitochondrial reactive oxygen species as signal-transducing messengers. Biochemistry.

[52] Caldon C.E. (2014). Estrogen signaling and the DNA damage response in hormone dependent breast cancers. Front. Oncol..

[53] Bhardwaj P., Au C.C., Benito-Martin A., Ladumor H., Oshchepkova S., Moges R., Brown K.A. (2019). Estrogens and breast cancer: Mechanisms involved in obesity-related development, growth and progression. J. Steroid Biochem. Mol. Biol..

[54] Fernandez S.V., Russo I.H., Russo J. (2006). Estradiol and its metabolites 4-hydroxyestradiol and 2-hydroxyestradiol induce mutations in human breast epithelial cells. Int. J. Cancer.

[55] Cavalieri E., Chakravarti D., Guttenplan J., Hart E., Ingle J., Jankowiak R., Muti P., Rogan E., Russo J., Santen R. (2006). Catechol estrogen quinones as initiators of breast and other human cancers: Implications for biomarkers of susceptibility and cancer prevention. Biochim. Biophys. Acta.

[56] Savage K.I., Matchett K.B., Barros E.M., Cooper K.M., Irwin G.W., Gorski J.J., Orr K.S., Vohhodina J., Kavanagh J.N., Madden A.F. (2014). BRCA1 deficiency exacerbates estrogen-induced DNA damage and genomic instability. Cancer Res..

[57] Alsudairi H.N., Alrasheed A.T., Dvornyk V. (2021). Estrogens and uterine fibroids: An integrated view. Res. Results Biomed..

[58] Yager J.D., Davidson N.E. (2006). Estrogen carcinogenesis in breast cancer. N. Engl. J. Med..

[59] Figueiredo J., da Cruz E., Silva O.A., Fardilha M. (2014). Protein phosphatase 1 and its complexes in carcinogenesis. Curr. Cancer Drug Targets.

[60] Rehman A.U., Najafi M., Kambouris M., Al-Gazali L., Makrythanasis P., Rad A., Maroofian R., Rajab A., Stark Z., Hunter J.V. (2019). Biallelic loss of function variants in PPP1R21 cause a neurodevelopmental syndrome with impaired endocytic function. Hum. Mutat..

[61] Yakirevich E., Resnick M.B., Mangray S., Wheeler M., Jackson C.L., Lombardo K.A., Lee J., Kim K.M., Gill A.J., Wang K. (2016). Oncogenic ALK Fusion in Rare and Aggressive Subtype of Colorectal Adenocarcinoma as a Potential Therapeutic Target. Clin. Cancer Res..

[62] Lu Y., Kweon S.S., Tanikawa C., Jia W.H., Xiang Y.B., Cai Q., Zeng C., Schmit S.L., Shin A., Matsuo K. (2019). Large-Scale Genome-Wide Association Study of East Asians Identifies Loci Associated with Risk for Colorectal Cancer. Gastroenterology.

[63] Sun Y.W., Chen K.M., Imamura Kawasawa Y., Salzberg A.C., Cooper T.K., Caruso C., Aliaga C., Zhu J., Gowda K., Amin S. (2017). Hypomethylated Fgf3 is a potential biomarker for early detection of oral cancer in mice treated with the tobacco carcinogen dibenzo[def,p]chrysene. PLoS ONE.

[64] Panebianco F., Nikitski A.V., Nikiforova M.N., Kaya C., Yip L., Condello V., Wald A.I., Nikiforov Y.E., Chiosea S.I. (2019). Characterization of thyroid cancer driven by known and novel ALK fusions. Endocr. Relat. Cancer.

[65] Velmurugan K.R., Varghese R.T., Fonville N.C., Garner H.R. (2017). High-depth, high-accuracy microsatellite genotyping enables precision lung cancer risk classification. Oncogene.

[66] Kang J., Zhang X.C., Chen H.J., Zhong W.Z., Xu Y., Su J., Zhou Q., Tu H.Y., Wang Z., Xu C.R. (2020). Complex ALK Fusions Are Associated with Better Prognosis in Advanced Non-Small Cell Lung Cancer. Front. Oncol..

[67] Zhao L., Nathenson M.J., Nowak J.A., Fairweather M., Hornick J.L. (2020). ALK rearrangement in a gastrointestinal stromal tumour of the small bowel. Histopathology.

[68] Wu Q., Hu Q., Hai Y., Li Y., Gao Y. (2023). METTL13 facilitates cell growth and metastasis in gastric cancer via an eEF1A/HN1L positive feedback circuit. J. Cell Commun. Signal..

[69] Cebrià-Costa J.P., Pascual-Reguant L., Gonzalez-Perez A., Serra-Bardenys G., Querol J., Cosín M., Verde G., Cigliano R.A., Sanseverino W., Segura-Bayona S. (2020). LOXL2-mediated H3K4 oxidation reduces chromatin accessibility in triple-negative breast cancer cells. Oncogene.

[70] Horvath A., Pakala S.B., Mudvari P., Reddy S.D., Ohshiro K., Casimiro S., Pires R., Fuqua S.A., Toi M., Costa L. (2013). Novel insights into breast cancer genetic variance through RNA sequencing. Sci. Rep..

[71] Jian Y., Kong L., Xu H., Shi Y., Huang X., Zhong W., Huang S., Li Y., Shi D., Xiao Y. (2022). Protein phosphatase 1 regulatory inhibitor subunit 14C promotes triple-negative breast cancer progression via sustaining inactive glycogen synthase kinase 3 beta. Clin. Transl. Med..

[72] Ruth K.S., Day F.R., Tyrrell J., Thompson D.J., Wood A.R., Mahajan A., Beaumont R.N., Wittemans L., Martin S., Busch A.S. (2020). Using human genetics to understand the disease impacts of testosterone in men and women. Nat. Med..

[73] Martin S., Cule M., Basty N., Tyrrell J., Beaumont R.N., Wood A.R., Frayling T.M., Sorokin E., Whitcher B., Liu Y. (2021). Genetic Evidence for Different Adiposity Phenotypes and Their Opposing Influences on Ectopic Fat and Risk of Cardiometabolic Disease. Diabetes.

[74] Haas C.B., Hsu L., Lampe J.W., Wernli K.J., Lindström S. (2022). Cross-ancestry Genome-wide Association Studies of Sex Hormone Concentrations in Pre- and Postmenopausal Women. Endocrinology.

[75] Yengo L., Vedantam S., Marouli E., Sidorenko J., Bartell E., Sakaue S., Graff M., Eliasen A.U., Jiang Y., Raghavan S. (2022). A saturated map of common genetic variants associated with human height. Nature.

[76] Kichaev G., Bhatia G., Loh P.R., Gazal S., Burch K., Freund M.K., Schoech A., Pasaniuc B., Price A.L. (2019). Leveraging Polygenic Functional Enrichment to Improve GWAS Power. Am. J. Hum. Genet..

[77] Zhu Z., Guo Y., Shi H., Liu C.L., Panganiban R.A., Chung W., O’Connor L.J., Himes B.E., Gazal S., Hasegawa K. (2020). Shared genetic and experimental links between obesity-related traits and asthma subtypes in UK Biobank. J. Allergy Clin. Immunol..

[78] Richardson T.G., Sanderson E., Palmer T.M., Ala-Korpela M., Ference B.A., Davey Smith G., Holmes M.V. (2020). Evaluating the relationship between circulating lipoprotein lipids and apolipoproteins with risk of coronary heart disease: A multivariable Mendelian randomisation analysis. PLoS Med..

[79] Zhang Q., Marioni R.E., Robinson M.R., Higham J., Sproul D., Wray N.R., Deary I.J., McRae A.F., Visscher P.M. (2018). Genotype effects contribute to variation in longitudinal methylome patterns in older people. Genome Med..

[80] Salimifard S., Masjedi A., Hojjat-Farsangi M., Ghalamfarsa G., Irandoust M., Azizi G., Mohammadi H., Keramati M.R., Jadidi-Niaragh F. (2020). Cancer associated fibroblasts as novel promising therapeutic targets in breast cancer. Pathol. Res. Pract..

[81] Hu D., Li Z., Zheng B., Lin X., Pan Y., Gong P., Zhuo W., Hu Y., Chen C., Chen L. (2022). Cancer-associated fibroblasts in breast cancer: Challenges and opportunities. Cancer Commun..

[82] Ma X.J., Dahiya S., Richardson E., Erlander M., Sgroi D.C. (2009). Gene expression profiling of the tumor microenvironment during breast cancer progression. Breast Cancer Res..

[83] Churnosov M., Abramova M., Reshetnikov E., Lyashenko I.V., Efremova O., Churnosova M., Ponomarenko I. (2022). Polymorphisms of hypertension susceptibility genes as a risk factors of preeclampsia in the Caucasian population of central Russia. Placenta.

[84] Pavlova N., Demin S., Churnosov M., Reshetnikov E., Aristova I., Churnosova M., Ponomarenko I. (2022). The Modifying Effect of Obesity on the Association of Matrix Metalloproteinase Gene Polymorphisms with Breast Cancer Risk. Biomedicines.

[85] Golovchenko I.O. (2023). Genetic determinants of sex hormone levels in endometriosis patients. Res. Results Biomed..

[86] Eliseeva N., Ponomarenko I., Reshetnikov E., Dvornyk V., Churnosov M. (2021). LOXL1 gene polymorphism candidates for exfoliation glaucoma are also associated with a risk for primary open-angle glaucoma in a Caucasian population from central Russia. Mol. Vis..

[87] Tikunova E., Ovtcharova V., Reshetnikov E., Dvornyk V., Polonikov A., Bushueva O., Churnosov M. (2017). Genes of tumor necrosis factors and their receptors and the primary open angle glaucoma in the population of Central Russia. Int. J. Ophthalmol..

[88] Novakov V., Novakova O., Churnosova M., Sorokina I., Aristova I., Polonikov A., Reshetnikov E., Churnosov M. (2023). Intergenic Interactions of SBNO1, NFAT5 and GLT8D1 Determine the Susceptibility to Knee Osteoarthritis among Europeans of Russia. Life.

[89] Ohlsson C., Wallaschofski H., Lunetta K.L., Stolk L., Perry J.R., Koster A., Petersen A.K., Eriksson J., Lehtimäki T., Huhtaniemi I.T. (2011). Genetic determinants of serum testosterone concentrations in men. PLoS Genet..

[90] Fantus R.J., Na R., Wei J., Shi Z., Resurreccion W.K., Halpern J.A., Franco O., Hayward S.W., Isaacs W.B., Zheng S.L. (2021). Genetic Susceptibility for Low Testosterone in Men and Its Implications in Biology and Screening: Data from the UK Biobank. Eur. Urol. Open Sci..

[91] Harrison S., Davies N.M., Howe L.D., Hughes A. (2021). Testosterone and socioeconomic position: Mendelian randomization in 306,248 men and women in UK Biobank. Sci. Adv..

[92] Golovchenko O., Abramova M., Ponomarenko I., Reshetnikov E., Aristova I., Polonikov A., Dvornyk V., Churnosov M. (2020). Functionally significant polymorphisms of ESR1and PGR and risk of intrauterine growth restriction in population of Central Russia. Eur. J. Obstet. Gynecol. Reprod. Biol..

[93] Reshetnikov E., Ponomarenko I., Golovchenko O., Sorokina I., Batlutskaya I., Yakunchenko T., Dvornyk V., Polonikov A., Churnosov M. (2019). The VNTR polymorphism of the endothelial nitric oxide synthase gene and blood pressure in women at the end of pregnancy. Taiwan. J. Obstet. Gynecol..

[94] Abramova M., Churnosova M., Efremova O., Aristova I., Reshetnikov E., Polonikov A., Churnosov M., Ponomarenko I. (2022). Effects of pre-pregnancy over-weight/obesity on the pattern of association of hypertension susceptibility genes with preeclampsia. Life.

[95] Purcell S., Neale B., Todd-Brown K., Thomas L., Ferreira M.A., Bender D., Maller J., Sklar P., de Bakker P.I., Daly M.J. (2007). PLINK: A tool set for whole-genome association and population-based linkage analyses. Am. J. Hum. Genet..

[96] Che R., Jack J.R., Motsinger-Reif A.A., Brown C.C. (2014). An adaptive permutation approach for genome-wide association study: Evaluation and recommendations for use. BioData Min..

[97] Gauderman W., Morrison J. (2006). QUANTO 1.1: A Computer Program for Power and Sample Size Calculations Genetic–Epidemiology Studies. http://hydra.usc.edu/gxe.

[98] Calle M.L., Urrea V., Malats N., Van Steen K. (2010). Mbmdr: An R package for exploring gene-gene interactions associated with binary or quantitative traits. Bioinformatics.

[99] Golovchenko I., Aizikovich B., Golovchenko O., Reshetnikov E., Churnosova M., Aristova I., Ponomarenko I., Churnosov M. (2022). Sex Hormone Candidate Gene Polymorphisms Are Associated with Endometriosis. Int. J. Mol. Sci..

[100] Ivanova T., Churnosova M., Abramova M., Ponomarenko I., Reshetnikov E., Aristova I., Sorokina I., Churnosov M. (2023). Risk Effects of rs1799945 Polymorphism of the HFE Gene and Intergenic Interactions of GWAS-Significant Loci for Arterial Hypertension in the Caucasian Population of Central Russia. Int. J. Mol. Sci..

[101] Reshetnikova Y., Churnosova M., Stepanov V., Bocharova A., Serebrova V., Trifonova E., Ponomarenko I., Sorokina I., Efremova O., Orlova V. (2023). Maternal Age at Menarche Gene Polymorphisms Are Associated with Offspring Birth Weight. Life.

[102] Motsinger A.A., Ritchie M.D. (2006). Multifactor dimensionality reduction: An analysis strategy for modelling and detecting gene-gene interactions in human genetics and pharmacogenomics studies. Hum. Genom..

[103] Moore J.H., Gilbert J.C., Tsai C.T., Chiang F.T., Holden T., Barney N., White B.C. (2006). A flexible computational framework for detecting, characterizing, and interpreting statistical patterns of epistasis in genetic studies of human disease susceptibility. J. Theor. Biol..

[104] Ivanova T., Churnosova M., Abramova M., Plotnikov D., Ponomarenko I., Reshetnikov E., Aristova I., Sorokina I., Churnosov M. (2023). Sex-Specific Features of the Correlation between GWAS-Noticeable Polymorphisms and Hypertension in Europeans of Russia. Int. J. Mol. Sci..

[105] Minyaylo O., Ponomarenko I., Reshetnikov E., Dvornyk V., Churnosov M. (2021). Functionally significant polymorphisms of the MMP-9 gene are associated with peptic ulcer disease in the Caucasian population of Central Russia. Sci. Rep..

[106] Polonikov A.V., Klyosova E.Y., Azarova I.E. (2021). Bioinformatic tools and internet resources for functional annotation of polymorphic loci detected by genome wide association studies of multifactorial diseases (review). Res. Results Biomed..

[107] Polonikov A., Rymarova L., Klyosova E., Volkova A., Azarova I., Bushueva O., Bykanova M., Bocharova I., Zhabin S., Churnosov M. (2019). Matrix metalloproteinases as target genes for gene regulatory networks driving molecular and cellular pathways related to a multistep pathogenesis of cerebrovascular disease. J. Cell Biochem..

[108] Sirotina S., Ponomarenko I., Kharchenko A., Bykanova M., Bocharova A., Vagaytseva K., Stepanov V., Churnosov M., Solodilova M., Polonikov A. (2018). A Novel Polymorphism in the Promoter of the *CYP4A11* Gene Is Associated with Susceptibility to Coronary Artery Disease. Dis. Markers.

[109] Ivanova T.A. (2024). Sex-specific features of interlocus interactions determining susceptibility to hypertension. Res. Results Biomed..

[110] GTEx Consortium (2020). The GTEx Consortium atlas of genetic regulatory effects across human tissues. Science.

[111] Zheng Z., Huang D., Wang J., Zhao K., Zhou Y., Guo Z., Zhai S., Xu H., Cui H., Yao H. (2020). QTLbase: An Integrative Resource for Quantitative Trait Loci across Multiple Human 846 Molecular Phenotypes. Nucleic Acids Res..

[112] Ward L.D., Kellis M. (2016). HaploReg v4: Systematic mining of putative causal variants, cell types, regulators and target genes for human complex traits and disease. Nucleic Acids Res..

[113] Zhang S., He Y., Liu H., Zhai H., Huang D., Yi X., Dong X., Wang Z., Zhao K., Zhou Y. (2019). regBase: Whole genome base-wise aggregation and functional prediction for human non-coding regulatory variants. Nucleic Acids Res..

[114] Kumar P., Henikoff S., Ng P.C. (2009). Predicting the effects of coding non-synonymous variants on protein function using the SIFT algorithm. Nat. Protoc..

[115] Szklarczyk D., Kirsch R., Koutrouli M., Nastou K., Mehryary F., Hachilif R., Gable A.L., Fang T., Doncheva N.T., Pyysalo S. (2023). The STRING database in 2023: Protein-protein association networks and functional enrichment analyses for any sequenced genome of interest. Nucleic Acids Res..

[116] Adzhubei I., Jordan D.M., Sunyaev S.R. (2013). Predicting functional effect of human missense mutations using PolyPhen-2. Curr. Protoc. Hum. Genet..

[117] Gene Ontology Consortium (2021). The Gene Ontology resource: Enriching a GOld mine. Nucleic Acids Res..

