# Supplementary material for: Sex-Hormone-Binding Globulin Gene Polymorphisms and Breast Cancer Risk in Caucasian Women of Russia
Source: Int J Mol Sci. 2024 Feb 11;25(4):2182. doi: 10.3390/ijms25042182 (PMC10888713; doi:10.3390/ijms25042182)
Supplement: Supplementary file 1 [file ijms-25-02182-s001.zip › =Suppl table 1.pdf]

Supplementary table 1

The allele and genotype frequencies of the studied SNPs in the breast cancer and control groups

Chr	SNP	Gene	Minor allele	Major allele	Minor allele frequency	Number of the studied chromosomes	Genotype distribution*	H_o	H_e	P_{HWE}
Breast cancer patients (n=358)										
1	rs17496332	*PRMT6*	G	A	0.355	704	51/148/153	0.421	0.458	0.131
2	rs780093	*GCKR*	T	C	0.394	706	46/186/121	0.527	0.477	0.059
2	rs10454142	*PPP1R21*	C	T	0.348	696	36/170/142	0.489	0.454	0.193
7	rs3779195	*BAIAP2L1*	A	T	0.185	688	9/109/226	0.317	0.301	0.472
8	rs440837	*ZBTB10*	G	A	0.229	700	22/116/212	0.331	0.353	0.288
10	rs7910927	*JMJD1C*	T	G	0.477	708	76/186/92	0.525	0.499	0.340
12	rs4149056	*SLCO1B1*	C	T	0.208	706	13/121/219	0.343	0.330	0.521
15	rs8023580	*NR2F2*	C	T	0.264	708	20/147/187	0.415	0.389	0.221
17	rs12150660	*SHBG*	T	G	0.251	708	23/132/199	0.373	0.376	0.888
Control group (n=1140)										
1	rs17496332	*PRMT6*	G	A	0.369	2140	152/486/432	0.454	0.466	0.431
2	rs780093	*GCKR*	T	C	0.405	2184	184/516/392	0.473	0.482	0.530
2	rs10454142	*PPP1R21*	C	T	0.314	2152	107/462/507	0.429	0.431	0.888
7	rs3779195	*BAIAP2L1*	A	T	0.176	2154	44/292/741	0.271	0.291	0.035
8	rs440837	*ZBTB10*	G	A	0.243	2116	57/401/600	0.379	0.368	0.404
10	rs7910927	*JMJD1C*	T	G	0.496	2184	265/554/273	0.507	0.500	0.672
12	rs4149056	*SLCO1B1*	C	T	0.224	2064	54/355/623	0.344	0.348	0.721
15	rs8023580	*NR2F2*	C	T	0.286	2172	101/420/565	0.387	0.409	0.075
17	rs12150660	*SHBG*	T	G	0.252	2196	81/391/626	0.356	0.377	0.078

Note: * minor allele homozygotes / heterozygotes / major allele homozygotes
