# Supplementary material for: Sex-Hormone-Binding Globulin Gene Polymorphisms and Breast Cancer Risk in Caucasian Women of Russia
Source: Int J Mol Sci. 2024 Feb 11;25(4):2182. doi: 10.3390/ijms25042182 (PMC10888713; doi:10.3390/ijms25042182)
Supplement: Supplementary file 1 [file ijms-25-02182-s001.zip › =Suppl table 15.pdf]

Supplementary table 15

The GWAS data about associations of the studied candidate gene polymorphisms with the circulating SHBG and other sex hormone concentrations

SNP, gene	Chromosome position (hg38)	Phenotype	Association (significance) (affected allele)	Reference
rs17496332 *PRMT6*	1p13.3 (107003753)	SHBG	β=-0.028 ($p=1\times10^{-11}$) (A)	[23]
rs780093 *GCKR*	2p23.3 (27519736)	SHBG	β=-0.032 ($p=2\times10^{-16}$) (T)	[23]
rs10454142 *FOXN2*	2p16.3 (48419260)	SHBG	β=0.026 ($p=1\times10^{-7}$) (T)	[23]
rs3779195 *BAIAP2L1*	7q21.3 (98364050)	SHBG	β=-0.033 ($p=3\times10^{-8}$) (A)	[23]
		SHBG (women, pre-menopause)	β=-2.41 ($p=9\times10^{-09}$) (A)	[92]
rs440837 *ZBTB10*	8q21.13 (80549739)	SHBG	β=-0.030 ($p=3\times10^{-9}$) (A)	[23]
		SHBG (women, post-menopause)	β=1.43 ($p=1\times10^{-12}$) (G)	[92]
		SHBG (men)	β=0.57 ($p=8\times10^{-9}$) (G)	[92]
rs7910927 *JMJD1C*	10q21.3 (63379150)	SHBG	β=-0.048 ($p=6\times10^{-35}$) (T)	[23]
rs4149056 *SLCO1B1*	12p12.1 (21178615)	SHBG	β=0.029 ($p=2\times10^{-8}$) (T)	[23]
		low testosterone levels (men)	OR=1.14 ($p=7\times10^{-16}$) (C)	[91]
		testosterone (women)	β=0.028 ($p=5\times10^{-10}$) (C)	[73]
		SHBG (women)	β=-0.065 ($p =5\times10^{-48}$) (C)	[73]
		SHBG (women, pre-menopause)	β=-0.062 ($p=8\times10^{-11}$) (C)	[73]
		SHBG (women, post-menopause)	β=-0.079 ($p=7\times10^{-34}$) (C)	[73]
		bioavailable testosterone (women)	β=0.02 ($p=2\times10^{-16}$) (C)	[92]
		SHBG (men)	β=-1.23 ($p=7\times10^{-29}$) (C)	[92]
		SHBG (women)	β=0.030 ($p=1\times10^{-73}$) (T)	[71]
		SHBG (men)	β=0.032 ($p=6\times10^{-99}$) (T)	[71]
		total testosterone (women)	β=-0.029 ($p=1\times10^{-14}$) (T)	[71]

		bioavailable testosterone (women)	β=-0.043 ($p=3\times10^{-35}$) (T)	[71]
		total testosterone (men)	β=0.054 ($p=1\times10^{-39}$) (T)	[71]
rs8023580 *PPP1R21*	15q26.2 (96165062)	SHBG	β=-0.03 ($p=8\times10^{-12}$) (T)	[23]
		low testosterone levels (men)	OR=1.13 ($p=1\times10^{-19}$) (T)	[91]
rs12150660 *SHBG*	17p13.1 (7618597)	SHBG	β=0.103 ($p=2\times10^{-106}$) (T)	[23]
		SHBG (women)	β=6.14 ($p=1\times10^{-300}$) (T)	[92]
		SHBG (men)	β=3.9 ($p=2\times10^{-75}$) (T)	[90]
		total testosterone (men)	β=31.8 ($p=1\times10^{-41}$) (T)	[90]
