# Supplementary material for: Sex-Hormone-Binding Globulin Gene Polymorphisms and Breast Cancer Risk in Caucasian Women of Russia
Source: Int J Mol Sci. 2024 Feb 11;25(4):2182. doi: 10.3390/ijms25042182 (PMC10888713; doi:10.3390/ijms25042182)
Supplement: Supplementary file 1 [file ijms-25-02182-s001.zip › =Suppl table 2.pdf]

Supplementary table 2

Genotype combinations associated with breast cancer*

Model	N	Genotype combinations	*beta*	P	Risk, High /Low
Two-order interaction models					
1	1	rs4149056-TC *SLCO1B1*- rs10454142-TC *PPP1R21*	0.552	0.006	H
	2	rs4149056-TT *SLCO1B1*- rs10454142-CC *PPP1R21*	0.595	0.047	H
	3	rs4149056-CC *SLCO1B1*- rs10454142-CC *PPP1R21*	1.662	0.013	H
2	4	rs440837-AA *ZBTB10* - rs10454142-TT *PPP1R21*	-0.333	0.039	L
	5	rs440837-AA *ZBTB10* - rs10454142-TC *PPP1R21*	0.344	0.031	H
	6	rs440837-AA *ZBTB10* - rs10454142-CC *PPP1R21*	0.782	0.006	H
	7	rs440837-GG *ZBTB10* - rs10454142-CC *PPP1R21*	2.003	0.009	H
Three-order interaction models					
1	1	rs7910927-GT *JMJD1C* - rs440837-AA *ZBTB10* - rs10454142-CC *PPP1R21*	1.586	0.00002	H
	2	rs7910927-GT *JMJD1C* - rs440837-GG *ZBTB10* - rs10454142-CC *PPP1R21*	2.819	0.016	H
2	3	rs4149056-TC *SLCO1B1* - rs440837-AA *ZBTB10* - rs10454142-TC *PPP1R21*	0.801	0.0009	H
	4	rs4149056-TT *SLCO1B1* - rs440837-AA *ZBTB10* - rs10454142-CC *PPP1R21*	0.878	0.012	H
	5	rs4149056-CC *SLCO1B1* - rs440837-AA *ZBTB10* - rs10454142-CC *PPP1R21*	3.756	0.016	H
Four-order interaction models					
1	1	rs4149056-TT *SLCO1B1* - rs440837-AA *ZBTB10* - rs10454142-CC *PPP1R21* - rs780093-CC *GCKR*	2.255	0.005	H
	2	rs4149056-TT *SLCO1B1* - rs440837-GG *ZBTB10* - rs10454142-TT *PPP1R21* - rs780093-CT *GCKR*	1.290	0.050	H
	3	rs4149056-TC *SLCO1B1* - rs440837-AA *ZBTB10* - rs10454142-TC *PPP1R21* - rs780093-CT *GCKR*	1.204	0.0002	H
	4	rs4149056-CC *SLCO1B1*-rs440837-AA *ZBTB10* - rs10454142-CC *PPP1R21* - rs780093-CT *GCKR*	3.756	0.016	H
	5	rs4149056-TC *SLCO1B1* - rs440837-AA *ZBTB10* - rs10454142-TC *PPP1R21* - rs780093-TT *GCKR*	1.356	0.017	H
2	6	rs8023580-TC *NR2F2*- rs7910927-GG *JMJD1C* - rs10454142-TC *PPP1R21* - rs17496332-AG *PRMT6*	1.200	0.021	H
	7	rs8023580-TT *NR2F2*- rs7910927-GT *JMJD1C* - rs10454142-CC *PPP1R21* - rs17496332-AA *PRMT6*	1.421	0.005	H
	8	rs8023580-TC *NR2F2*- rs7910927-GT *JMJD1C* - rs10454142-CC *PPP1R21* - rs17496332-AA *PRMT6*	1.649	0.004	H
	9	rs8023580-TC *NR2F2*- rs7910927-TT *JMJD1C* - rs10454142-TT *PPP1R21* - rs17496332-AG *PRMT6*	-1.431	0.045	L
	10	rs8023580-CC *NR2F2*- rs7910927-GT *JMJD1C* - rs10454142-TC *PPP1R21* - rs17496332-AG *PRMT6*	1.517	0.050	H
3	11	rs7910927-GT *JMJD1C*-rs10454142-TC *PPP1R21*-rs780093-CC *GCKR* - rs17496332-AA *PRMT6*	-1.827	0.007	L

	12	rs7910927-GT *JMJD1C*-rs10454142-CC *PPP1R21*-rs780093-CC *GCKR* - rs17496332-AA *PRMT6*	3.926	0.0003	H
	13	rs7910927-GG *JMJD1C* - rs10454142-TC *PPP1R21* - rs780093-CC *GCKR* - rs17496332-AA *PRMT6*	1.732	0.027	H
	14	rs7910927-GT *JMJD1C* - rs10454142-CC *PPP1R21* - rs780093-CT *GCKR* - rs17496332-AA *PRMT6*	1.022	0.046	H
	15	rs7910927-TT *JMJD1C* - rs10454142-TC *PPP1R21* - rs780093-CT *GCKR* - rs17496332-AA *PRMT6*	-0.677	0.049	L
	16	rs7910927-GT *JMJD1C* - rs10454142-TT *PPP1R21* - rs780093-TT *GCKR* - rs17496332-AG *PRMT6*	2.600	0.027	H
	17	rs7910927-GT *JMJD1C* - rs10454142-TC *PPP1R21* - rs780093-CT *GCKR* - rs17496332-GG *PRMT6*	2.520	0.004	H
	18	rs7910927-GG *JMJD1C* - rs10454142-TT *PPP1R21* - rs780093-CC *GCKR* - rs17496332-GG *PRMT6*	1.252	0.039	H
Five-order interaction models					
1	1	rs7910927-GT *JMJD1C* - rs3779195-TT *BAIAP2L1* - rs10454142-TC *PPP1R21* - rs780093-CC *GCKR* - rs17496332-AA *PRMT6*	-1.827	0.007	L
	2	rs7910927-GT *JMJD1C*- rs3779195-TA *BAIAP2L1* - rs10454142-CC *PPP1R21* - rs780093-CT *GCKR* - rs17496332-AA *PRMT6*	3.926	0.0003	H
	3	rs7910927-TT *JMJD1C* - rs3779195-TT *BAIAP2L1* - rs10454142-CC *PPP1R21* - rs780093-TT *GCKR* - rs17496332-AA *PRMT6*	1.732	0.027	H
	4	rs7910927-GG *JMJD1C*- rs3779195-TT *BAIAP2L1* - rs10454142-TC *PPP1R21* - rs780093-CC *GCKR* - rs17496332-AG *PRMT6*	1.022	0.046	H
	5	rs7910927-GT *JMJD1C* - rs3779195-TT *BAIAP2L1* - rs10454142-TT *PPP1R21* - rs780093-CT *GCKR* - rs17496332-AG *PRMT6*	-0.677	0.049	L
	6	rs7910927-GG *JMJD1C*- rs3779195-TA *BAIAP2L1* - rs10454142-CC *PPP1R21* - rs780093-CT *GCKR* - rs17496332-AG *PRMT6*	2.600	0.027	H
	7	rs7910927-GT *JMJD1C* - rs3779195-TA *BAIAP2L1* - rs10454142-TT *PPP1R21* - rs780093-CC *GCKR* - rs17496332-GG *PRMT6*	2.520	0.004	H
	8	rs7910927-GT *JMJD1C* - rs3779195-TT *BAIAP2L1* - rs10454142-TC *PPP1R21* - rs780093-CC *GCKR* - rs17496332-GG *PRMT6*	1.252	0.039	H
	9	rs7910927-GG *JMJD1C* - rs3779195-TT *BAIAP2L1* - rs10454142-TT *PPP1R21* - rs780093-TT *GCKR* - rs17496332-GG *PRMT6*	3.674	0.024	H
2	10	rs8023580-TC *NR2F2* - rs7910927-GT *JMJD1C* - rs10454142-CC *PPP1R21* - rs780093-CC *GCKR* - rs17496332-AA *PRMT6*	2.072	0.023	H
	11	rs8023580-TT *NR2F2* - rs7910927-GT *JMJD1C* - rs10454142-CC *PPP1R21* - rs780093-CT *GCKR* - rs17496332-AA *PRMT6*	1.883	0.012	H
	12	rs8023580-TT *NR2F2* - rs7910927-GG *JMJD1C* - rs10454142-TC *PPP1R21* - rs780093-CC *GCKR* - rs17496332-AG *PRMT6*	1.369	0.031	H
	13	rs8023580-TC *NR2F2* - rs7910927-GG *JMJD1C* - rs10454142-TC *PPP1R21* - rs780093-CT *GCKR* - rs17496332-AG *PRMT6*	-2.202	0.048	L
	14	rs8023580-TC *NR2F2* - rs7910927-GT *JMJD1C* - rs10454142-CC *PPP1R21* - rs780093-CT *GCKR* - rs17496332-AG *PRMT6*	2.058	0.027	H

* Genotype combinations are derived from the interaction models obtained by the MB-MDR method and described in tables 1
